# Genetic subtraction profiling identifies genes essential for *Arabidopsis *reproduction and reveals interaction between the female gametophyte and the maternal sporophyte

**DOI:** 10.1186/gb-2007-8-10-r204

**Published:** 2007-10-03

**Authors:** Amal J Johnston, Patrick Meier, Jacqueline Gheyselinck, Samuel EJ Wuest, Michael Federer, Edith Schlagenhauf, Jörg D Becker, Ueli Grossniklaus

**Affiliations:** 1 Institute of Plant Biology and Zürich-Basel Plant Science Center, Zollikerstrasse, University of Zürich, CH-8008 Zürich, Switzerland; 2 Centro de Biologia do Desenvolvimento, Instituto Gulbenkian de Ciência, Rua da Quinta Grande, PT-2780-156 Oeiras, Portugal; 3 Current address: Institute of Plant Sciences and Zürich-Basel Plant Science Center, ETH Zürich, Universitätstrasse, CH-8092 Zürich, Switzerland

## Abstract

**Background:**

The embryo sac contains the haploid maternal cell types necessary for double fertilization and subsequent seed development in plants. Large-scale identification of genes expressed in the embryo sac remains cumbersome because of its inherent microscopic and inaccessible nature. We used genetic subtraction and comparative profiling by microarray between the *Arabidopsis thaliana *wild-type and a sporophytic mutant lacking an embryo sac in order to identify embryo sac expressed genes in this model organism. The influences of the embryo sac on the surrounding sporophytic tissues were previously thought to be negligible or nonexistent; we investigated the extent of these interactions by transcriptome analysis.

**Results:**

We identified 1,260 genes as embryo sac expressed by analyzing both our dataset and a recently reported dataset, obtained by a similar approach, using three statistical procedures. Spatial expression of nine genes (for instance a central cell expressed *trithorax*-like gene, an egg cell expressed gene encoding a kinase, and a synergid expressed gene encoding a permease) validated our approach. We analyzed mutants in five of the newly identified genes that exhibited developmental anomalies during reproductive development. A total of 527 genes were identified for their expression in ovules of mutants lacking an embryo sac, at levels that were twofold higher than in the wild type.

**Conclusion:**

Identification of embryo sac expressed genes establishes a basis for the functional dissection of embryo sac development and function. Sporophytic gain of expression in mutants lacking an embryo sac suggests that a substantial portion of the sporophytic transcriptome involved in carpel and ovule development is, unexpectedly, under the indirect influence of the embryo sac.

## Abstract 2

Genetic subtraction and expression profiling of wild-type *Arabidopsis *and a sporophytic mutant lacking an embryo sac identified 1,260 genes expressed in the embryo sac; a total of 527 genes were identified for their expression in ovules of mutants lacking an embryo sac.

## Background

The life cycle of plants alternates between diploid (sporophyte) and haploid (male and female gametophytes) generations. The multicellular gametophytes represent the haploid phase of the life cycle between meiosis and fertilization, during which the gametes are produced through mitotic divisions. Double fertilization is unique to flowering plants; the female gametes, namely the haploid egg cell and the homo-diploid central cell, are fertilized by one sperm cell each. Double fertilization produces a diploid embryo and a triploid endosperm, which are the two major constituents of the developing seed [1]. The egg, the central cell, and two accessory cell types (specifically, two synergid cells and three antipodal cells) are contained in the embryo sac, also known as the female gametophyte or megagametophyte, which is embedded within the maternal tissues of the ovule. As a carrier of maternal cell types required for fertilization, the embryo sac provides an interesting model in which to study a variety of developmental aspects relating to cell specification, cell polarity, signaling, cell differentiation, double fertilization, genomic imprinting, and apomixis [1-3].

Out of the 28,974 predicted open reading frames of *Arabidopsis thaliana*, a few thousand genes are predicted to be involved in embryo sac development [1,4]. These genes can be grouped into two major classes: genes that are necessary during female gametogenesis and genes that impose maternal effects through the female gametophyte, and thus play essential roles for seed development. To date, loss-of-function mutational analyses have identified just over 100 genes in *Arabidopsis *that belong to these two classes [5-14]. However, only a small number of genes have been characterized in depth. Cell cycle genes (for instance, *PROLIFERA*, *APC2 *[*ANAPHASE PROMOTING COMPLEX 2*], *NOMEGA*, and *RBR1 *[*RETINOBLASTOMA RELATED 1*]), transcription factors (for instance, *MYB98 *and *AGL80 *[*AGAMOUS-LIKE-80*]), and others (including *CKI1 *[*CYTOKININ INDEPENDENT 1*], *GFA2 *[*GAMETOPHYTIC FACTOR 2*], *SWA1 *[*SLOW WALKER 1*] and *LPAT2 *[*LYSOPHOSPHATIDYL ACYLTRANSFERASE 2*]) are essential during embryo sac development [6,15-23]. Maternal effect genes include those of the *FIS *(*FERTILIZATION INDEPENDENT SEED*) class and many others that are less well characterized [9,13,24]. *FIS *genes are epigenetic regulators of the *Polycomb *group and control cell proliferation during endosperm development and embryogenesis [7,10,12,25,26]. Ultimately, the molecular components of cell specification and cell differentiation during megagametogenesis and double fertilization remain largely unknown, and alternate strategies are required for a high-throughput identification of candidate genes expressed during embryo sac development.

Although transcriptome profiling of *Arabidopsis *floral organs [27,28], whole flowers and seed [29], and male gametophytes [30-33] have been reported in previous studies, large-scale identification of genes expressed during female gametophyte development remains cumbersome because of the microscopic nature of the embryo sac. Given the dearth of transcriptome data, we attempted to explore the *Arabidopsis *embryo sac transcriptome using genetic subtraction and microarray-based comparative profiling between the wild type and a sporophytic mutant, *coatlique *(*coa*), which lacks an embryo sac. Using such a genetic subtraction, genes whose transcripts were present in the wild type at levels higher than in *coa *could be regarded as embryo sac expressed candidate genes. While our work was in progress, Yu and coworkers [34] reported a similar genetic approach to reveal the identity of 204 genes expressed in mature embryo sacs. However, their analysis of the embryo sac transcriptome was not exhaustive because they used different statistical methodology in their data analysis. Thus, we combined their dataset with ours for statistical analyses using three statistical packages in order to explore the transcriptome more extensively. Here, we report the identity of 1,260 potentially embryo sac expressed genes, 8.6% of which were not found in tissue-specific sporophytic transcriptomes, suggesting selective expression in the embryo sac. Strong support for the predicted transcriptome was provided by the spatial expression pattern of 24 genes in embryo sac cells; 13 of them were previously identified as being expressed in the embryo sac by enhancer detectors or promoter-reporter gene fusions, and we could confirm the spatial expression of the corresponding transcripts by microarray analysis. In addition, we show embryo sac cell-specific expression for nine novel genes by *in situ *hybridization or reporter gene fusions. In order to elucidate the functional role of the identified genes, we sought to search for mutants affecting embryo sac and seed development by T-DNA mutagenesis. We describe the developmental anomalies evident in five mutants exhibiting lethality during female gametogenesis or seed development.

Genetic evidence suggests that the maternal sporophyte influences development of the embryo sac [1,35-37]. Because the carpel and sporophytic parts of the ovule develop normally in the absence of an embryo sac, it has been concluded that the female gametophyte does not influence gene expression in the surrounding tissue [2]. Our data clearly showed that 527 genes were over-expressed by at least twofold in the morphologically normal maternal sporophyte in two sporophytic mutants lacking an embryo sac. We confirm the gain of expression of 11 such genes in mutant ovules by reverse transcription polymerase chain reaction (RT-PCR). Spatial expression of five of these genes in carpel and ovule tissues of *coa *was confirmed by *in situ *hybridization, revealing that expression mainly in the carpel and ovule tissues is tightly correlated with the presence or absence of an embryo sac. In summary, our study provides two valuable datasets of the transcriptome of *Arabidopsis *gynoecia, comprising a total of 1,787 genes: genes that are expressed or enriched in the embryo sac and are likely function to control embryo sac and seed development; and a set of genes that are over-expressed in the maternal sporophyte in the absence of a functional embryo sac, revealing interactions between gametophytic and sporophytic tissues in the ovule and carpel.

## Results

**Figure 1:**
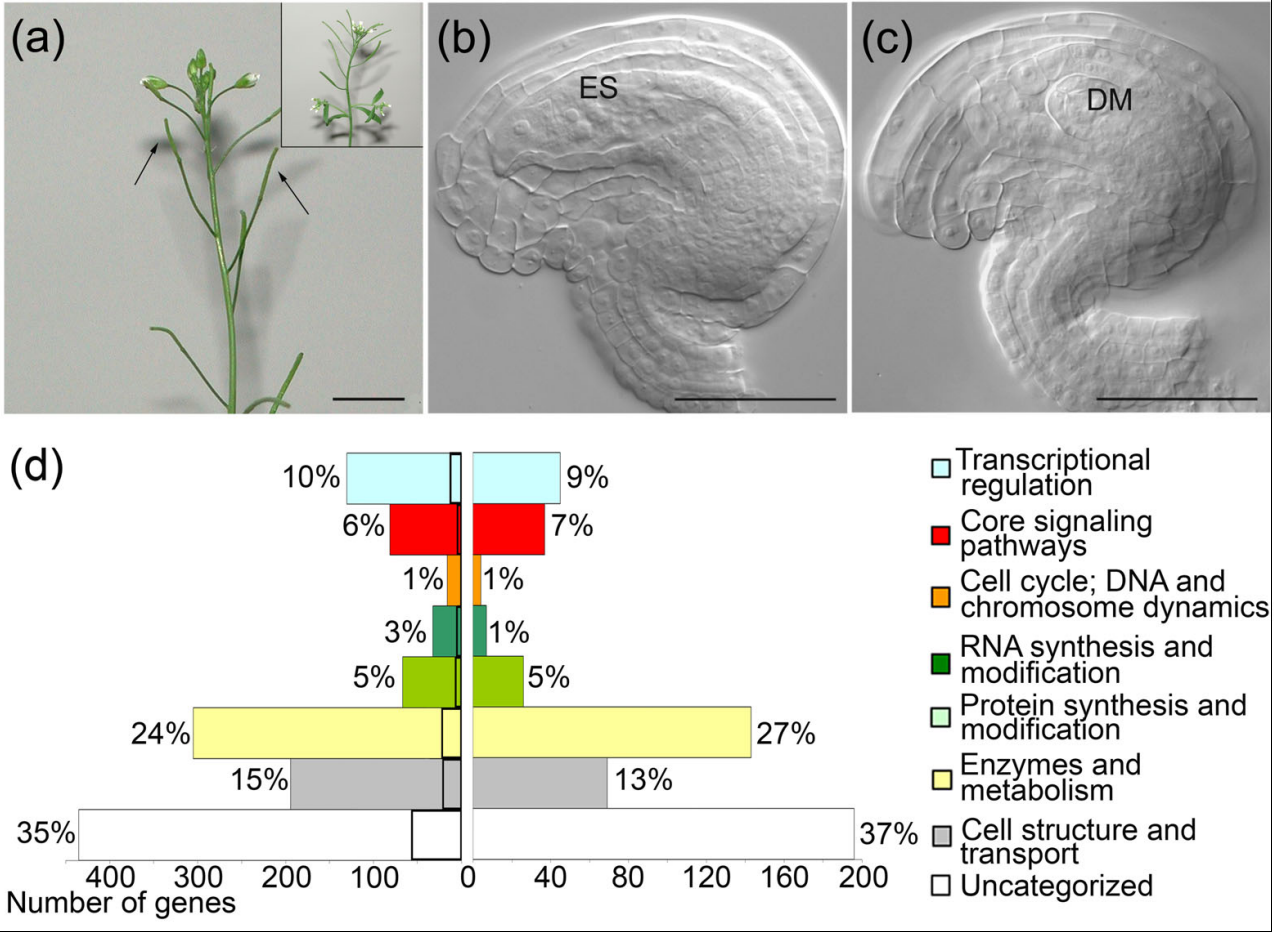
A genetic subtraction strategy for determination of the embryo sac transcriptome. **(a) **A branch of a *coatlique *(*coa*) showing undeveloped siliques. Arrows point to a small silique, which bears female sterile ovules inside the carpel (insert: wild-type L*er *branch). **(b) **Morphology of a mature wild-type ovule bearing an embryo sac (ES) before anthesis. **(c) **A functional embryo sac is absent in *coa *(degenerated megaspores [DM]). Note that the ovule sporophyte is morphologically equivalent to that of the wild type. **(d) **Functional categories of genes identified by a microarray-based comparison of *coa *and *sporocyteless *(*spl*; based on data from Yu and coworkers [34]) with the wild type. The embryo sac expressed transcriptome is shown to the left. Embryo sac expressed genes were grouped as preferentially expressed in the embryo sac if they were not detected in previous sporophytic microarrays [28]. The size of the specific transcriptome in each class is marked on each bar by a dark outline. Functional categories of genes that were identified as over-expressed in the sporophyte of *coa *and *spl *are shown to the right. Scale bars: 1 cm in panel a (2 cm in the insert of panel a), and 50 μm in panels b and c.

We intended to isolate genes that are expressed in the mature female gametophyte of *A. thaliana*, and are thus potentially involved in its development and function. To this end, the transcriptomes of the gynoecia from wild-type plants were compared with those of two sporophytic recessive mutants, namely *coatlique *(*coa*) and *sporocyteless *(*spl*), both of which lack a functional embryo sac. The *coa *mutant was isolated during transposon mutagenesis for its complete female sterility and partial male sterility in the homozygous state (Vielle-Calzada J-P, Moore JM, Grossniklaus U, unpublished data). Following tetrad formation three megaspores degenerated, producing one viable megaspore, but megagametogenesis was not initiated in *coa*. Despite the failure in embryo sac development, the integuments and endothelium in *coa *differentiated similar to wild-type ovules (Figure 1). In addition to our experiment with *coa*, we reanalyzed the dataset reported by Yu and coworkers [34], who used the *spl *mutant and corresponding wild type for a similar comparison. The *spl *mutant behaves both phenotypically and genetically very similar to *coa *[38]. The primary difference in the experimental set up between the present study and that conducted by Yu and coworkers [34] is that we did not dissect out the ovules from pistils, whereas Yu and coworkers extracted ovule samples by manual dissection from the carpel, which led to a lower dilution of 'contaminating' cells surrounding the embryo sac. However, our inclusion of intact pistils allowed us to elucidate the carpel-specific and ovule-specific effects controlled by the female gametophyte.

### Statistical issues on the microarray data analysis

To determine the embryo sac transcriptome, we used *coa *and wild-type pistil samples (late 11 to late 12 floral stages [39]) in three biologic replicates, and followed the Affymetrix standard procedures from cRNA synthesis to hybridization on the chip. Finally, raw microarray data from the *coa *and wild-type samples in triplicate were retrieved after scanning the *Arabidopsis *ATH1 'whole genome' chips, which represent 24,000 annotated genes, and they were subjected to statistical analyses. The normalized data were examined for their quality using cluster analysis [40]. There was strong positive correlation between samples within the three replicates of wild-type and *coa *(Pearson coefficients: *r *= 0.967 for for wild-type and *r *= 0.973 for *coa*). Therefore, the data were considered to be of good quality for further analyses. It was necessary to ensure that the arrays of both the wild type and *coa *did not differ in RNA quality and hybridization efficiency. The hybridization signal intensities of internal control gene probes were not significantly altered across the analysed arrays, hence assuring the reliability of the results (data not shown). The quality of data for the *spl *mutant and wild-type microarray was described previously [34]. Subsequently, differentially expressed genes were identified using three independent microarray data analysis software packages.

To identify genes that are expressed in the female gametophyte, we subtracted the transcriptomes of *coa *or *spl *from the corresponding wild type. Genes that were identified as being upregulated in wild-type gynoecia are candidates for female gametophytic expression, and genes highly expressed in *coa *and *spl *are probable candidates for gain-of-expression in the sporophyte of these mutants. However, this comparison was not straightforward because we were not in a position to compare the mere four cell types of the mature embryo sac with the same number of sporophytic cells. Whether using whole pistils or isolated ovules, a large excess of sporophytic cells surrounds the embryo sac. The contaminating cells originate from the ovule tissues such as endothelium, integuments and funiculus, or those surrounding the ovules such as stigma, style, transmitting tract, placenta, carpel wall and replum. Therefore, we anticipated that the transcript subtraction for embryo sac expression would suffer from high experimental noise. We examined the log transformed data points from the *coa *and *spl *datasets (with their corresponding wild-type data) in volcano plots. This procedure allows us to visualize the trade-offs between the fold change and the statistical significance. As we anticipated, the data points from the sporophytic gain outnumbered the embryo sac transcriptome data points on a high-stringency scale (data not shown). This problem of dilution in our data for embryo sac gene discovery was more pronounced in the *coa *dataset than that of *spl*, because we did not dissect out the ovules from the carpel. Therefore, we made the following decisions in analyzing the gametophytic data: to use advanced statistical packages that use different principles in their treatment of the data; and to set a lowest meaningful fold change in data comparison, in contrast to the usual twofold change as recommended in the literature.

In the recent past, many new pre-processing methods for Affymetrix GeneChip data have been developed, and there are conflicting reports about the performance of each algorithm [41-43]. Because there is no consensus about the most accurate analysis methods, contrasting methods can be combined for gene discovery [44]. We used the following three methods in data analyses: the microarray suite software (MAS; Affymetrix) and Genspring; the DNA Chip analyzer (dCHIP) package [45]; and GC robust multi-array average analysis (gcRMA) [46]. MAS uses a nonparametric statistical method in data analyses, whereas dCHIP uses an intensity modeling approach [47]. dCHIP removes outlier probe intensities, and reduces the between-replicate variation [48]. A more recent method, gcRMA uses a model-based background correction and a robust linear model to calculate signal intensities. Depending on the particular question to be addressed, one may wish to identify genes that are expressed in the embryo sac with the highest probability possible and to use a very stringent statistical treatment (for example, dCHIP), or one may wish to obtain the widest possible range of genes that are potentially expressed in the embryo sac and employ a less stringent method (for example, MAS). We did not wish to discriminate between the three methods in our analysis, and we provide data for all of them.

Although conventionally twofold change criteria have been followed in a number of microarray studies, it has been disputed whether fold change should be used at all to study differential gene expression (for review, see [49]). Based on studies correlating both microarray and quantitative RT-PCR data, it was suggested that genes exhibiting 1.4-fold change could be used reliably [50,51]. Tung and coworkers used a minimum fold change as low as 1.2 in order to identify differentially expressed genes in *Arabidopsis *pistils within specific cell types, and the results were spatially validated [52]. In order to make a decision on our fold change criterion in the data analysis, we examined the dataset for validation of embryo sac expressed genes that had previously been reported. We found that genes such as *CyclinA2;4 *(*coa *dataset) and *ORC2 *(*spl *dataset) were identified at a fold change of 1.28 (Additional data file 1). In addition, out of the 43 predicted genes at 1.28-fold change from *coa *and *spl *datasets, 33% were present in triplicate datasets from laser captured central cells (Wuest S, Vijverberg K, Grossniklaus U, unpublished data), independently confirming their expression in at least one cell of the embryo sac. Therefore, the baseline cut-off for subtraction was set at 1.28-fold in the wild type, and a total of 1,260 genes were identified as putative candidates for expression in the female gametophyte (Additional data files 2 and 3).

However, it must be noted that lowering the fold change potentially increases the incidence of false-positive findings. By setting the baseline to 1.28, we could predict that false discovery rates (FDRs) would range between 0.05% and 3.00%, based on dCHIP and gcRMA analyses (data not shown). Convincingly, we we able to observe 24 essential genes and 17 embryo sac expressed genes at a fold change range between 1.28 and 1.6 (Additional data files 1 and 4, and references therein). Moreover, our data on homology of candidate genes to expressed sequence tags (ESTs) from monocot embryo sacs will facilitate careful manual omission of false-positive findings. The usefulness of this approach is also demonstrated by the observation that 84% of the essential genes and genes validated for embryo sac expression (*n *= 51) present in our datasets exhibited homology to the monocot embryo sac ESTs. Therefore, our practical strategy of using a low fold change cut-off probably helped in identifying low-abundance signals, which would otherwise be ignored or handled in an *ad hoc *manner.

In contrast to the embryo sac datasets, we applied a more stringent twofold higher expression as a baseline for comparison of the mutant sporophyte with the wild type. This is because we had large amounts of sporophytic cells available for comparison. In all, 527 genes were identified as candidate genes for gain of sporophytic expression in *coa *and *spl *mutant ovules (Additional data file 5). Because the transcriptome identified by three independent statistical methods and the resultant overlaps were rather different in size for both the gametophytic and sporophytic datasets, we report all the data across the three methods (Additional data file 6). This approach is validated by the fact that candidate genes found using only one statistical method can indeed be embryo sac expressed (see Additional data file 7). Furthermore, only 8% of the validated genes (*n *= 51) were consistently identified by all three methods, demonstrating the need for independent statistical treatments (Additional data file 7). In short, our data analyses demonstrate the usefulness of employing different statistical treatments for microarray data.

Another practical consideration following our data analyses was the very limited overlap between *coa *and *spl *datasets. Although both mutants are genetically and phenotypically similar, the overlap is only 35 genes between the embryo sac datasets and 13 genes between the sporophytic datasets (Additional data files 2, 3, and 5). In light of the validation in expression for 12 genes from the *coa *dataset, which were not identified from the *spl *dataset, we suggest that the limited overlap is not merely due to experimental errors. It is likely that the embryo sac transcriptome is substantial (several thousands of genes [2]), and two independent experiments identified different subsets of the same transcriptome. This is apparent from our validation of expression for several genes, which were exclusively found in only one microarray dataset (Additional data file 1). In terms of the sporophytic gene expression, we have shown that three sporophytic genes initially identified only in the *spl *microarray dataset were indeed over-expressed in *coa *tissues (discussed below). In short, despite the limited overlap between datasets, both the embryo sac and sporophytic datasets will be very useful in elucidating embryo sac development and its control of sporophytic gene expression.

### Functional classification of the candidate genes

The genes identified as embryo sac expressed or over-expressed sporophytic candidates were grouped into eight functional categories based on a classification system reported previously [53] (Figure 1). The gene annotations were improved based on the Gene Ontology annotations available from 'The *Arabidopsis *Information Resource' (TAIR). The largest group in both gene datasets consisted of genes with unknown function (35% of embryo sac expressed genes and 37% of over-expressed sporophytic candidate genes), and the next largest was the class of metabolic genes (24% and 27%; Figure 1). Overall, both the gametophytic and sporophytic datasets comprised similar percentages of genes within each functional category (Figure 1). In both datasets, we found genes that are predicted to be involved in transport facilitation and cell wall biogenesis (15% of embryo sac expressed genes and 13% of over-expressed sporophytic candidate genes), transcriptional regulation (10% and 9%), signaling (7% and 6%), translation and protein fate (5% each), RNA synthesis and modification (3% and 1%), and cell cycle and chromosome dynamics (1% each).

### Validation of expression for known embryo sac-expressed genes

The efficacy of the comparative profiling approach used here was first confirmed by the presence of 18 genes that were previously identified as being expressed in the embryo sac (Additional data file 1). They included embryo sac expressed genes such as *PROLIFERA*, *PAB2 *and *PAB5 *(which encode poly-A binding proteins) and *MEDEA*, and genes with cell-specific expression such as central cell expressed *FIS2 *and *FWA*, synergid cell expressed *MYB98*, and antipodal cell expressed *AT1G36340 *(Additional data file 1 and references therein). Therefore, our comparative profiling approach potentially identified novel genes that could be expressed either throughout the embryo sac or in an expression pattern that is restricted to specific cell types.

### *In situ *hybridization and enhancer detector patterns confirm embryo sac expression of candidate genes

**Figure 2:**
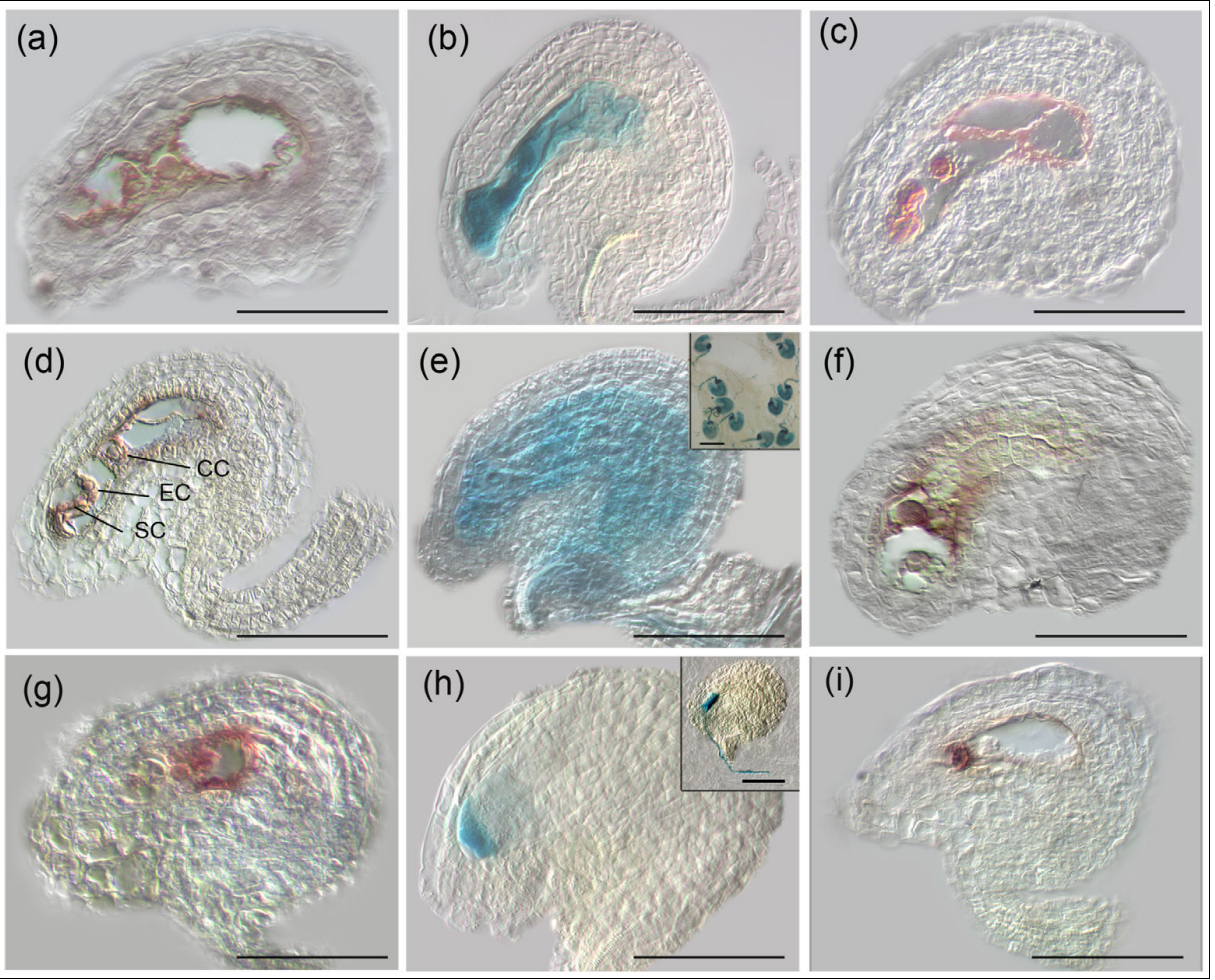
Confirmation of embryo sac expression for selected genes. Embryo sac expression of nine candidate genes is shown by *in situ *hybridization (panels a, c, d, f, g, and i) or histochemical reporter gene (GUS) analysis (b, e, and h). Illustrated is the *in situ *expression of broadly expressed genes: **(a) ***AT1G78940 *(encoding a protein kinase that is involved in regulation of cell cycle progression), **(c) ***AT5G40260 *(encoding a nodulin), and **(d) ***AT4G30590 *(encoding a plastocyanin). Also shown is the restricted expression of **(f) ***AT3G61740 *(encoding the *trithorax*-like protein ATX3), **(g) ***AT5G50915 *(encoding a TCP transcription factor), and **(i) ***AT5G60270 *(encoding a protein kinase). The corresponding sense control for panels a, b, c, d, f, g, and i did not show any detectable signal (data not shown). GUS staining: **(b) **an enhancer-trap line for *AT4G01970 *(encoding a galactosyltransferase) shows embryo sac expression, **(e) **a promoter-GUS line for *AT1G80370 *(encoding CYCLIN A2;4) shows a strong and specific expression in the embryo sac and endothelium (insert: shows several ovules at lower magnification), and **(h) **a promoter-GUS line for *AT1G28220 *(encoding the purine permease PUP3) shows synergid specific expression (insert; note the pollen-specific expression of *PUP3-GUS *when used as a pollen donor on a wild-type pistil). CC, central cell; EC, egg cell; SC, synergids. Scale bars: 50 μm in panels a to i; and 100 μm and 50 μm in the inserts of panels e and h, respectively.

In order to validate the spatial expression of candidate genes in the wild-type embryo sac, the six following genes were chosen for mRNA *in situ *hybridization on paraffin-embedded pistils: *AT5G40260 *(encoding nodulin; 1.99-fold) and *AT4G30590 *(encoding plastocyanin; 1.88-fold); *AT5G60270 *(encoding a receptor-like kinase; 1.56-fold) and *AT3G61740 *(encoding TRITHORAX-LIKE 3 [ATX3]; 1.47-fold); and *AT5G50915 *(encoding a TCP transcription factor; 1.36-fold) and *AT1G78940 *(encoding a protein kinase; 1.35-fold). Broad expression in all cells of the mature embryo sac was observed for genes *AT5G40260*, *AT4G30590*, *AT5G60270*, and *AT4G01970 *(Figure 2). The *trithorax *group gene *ATX3 *and *AT5G50915 *were predominantly expressed in the egg and the central cell, and the expression of the receptor-like kinase gene *AT5G60270 *was found to be restricted to the egg cell alone (Figure 2). In addition to the *in situ *hybridization experiments, we examined the expression of transgenes where specific promoters drive the expression of the bacterial *uidA *gene encoding β-galacturonidase (GUS) or in enhancer detector lines. We show that *CYCLIN A2;4 *(1.28-fold) and *AT4G01970 *(encoding a galactosyl-transferase; about 1.51-fold) were broadly expressed in the embryo sac, and that *PUP3 *(encoding a purine permease; 1.3-fold) was specifically expressed in the synergids (Figure 2). *CYCLIN A2;4 *appears to be expressed also in the endothelial layer surrounding the embryo sac (Figure 2e). Diffusion of GUS activity did not permit us to distinguish unambiguously embryo sac expression from endothelial expression. In short, both broader and cell type specific expression patterns in the embryo sac were observed for the nine candidate genes. Hence, we could validate the minimal fold change cut-off of 1.28 and the statistical methods employed in this study.

### Embryo sac enriched genes

Our strategic approach to exploring the embryo sac transcriptome was twofold: we aimed first to identify embryo sac expressed genes; second to describe the gametophyte enriched (male and female) transcriptome; and finally to define the embryo sac enriched (female only) transcriptome. Although the first category does not consider whether an embryo sac expressed gene is also expressed in the sporophyte, the second class of genes are grouped for their enriched expression in the male (pollen) and female gametophyte, but not in the sporophyte. The embryo sac enriched transcriptome is a subset of the gametophyte enriched transcriptome, wherein male gametophyte expressed genes are omitted. Of the embryo sac expressed genes, 32% were also present in the mature pollen transcriptome, and the vast majority (77%) were expressed in immature siliques as expected (Additional data files 2 and 3). Because large-scale female gametophytic cell expressed transcriptome data of *Arabidopsis *based on microarray or EST analyses are not yet available, we compared our data with the publicly available cell specific ESTs from maize and wheat by basic local alignment search tool (BLAST) analysis. Large-scale monocot ESTs are available only for the embryo sac and egg cells but not for the central cells (only 30 central cell derived ESTs from [54]). Therefore, we included the ESTs from immature endosperm cells at 6 days after pollination in the data comparison (Additional data file 8 and the references therein). Of our candidate genes, 38% were similar to the monocot embryo sac ESTs, 33% to the egg ESTs, and 53% to the central cell and endosperm ESTs (Additional data files 2 and 3).

Genes that were enriched in both the male and female gametophytes, or only in the embryo sac, were identified by subtracting these transcriptomes from a vast array of plant sporophytic transcriptomes of leaves, roots, whole seedlings, floral organs, pollen, and so on (Additional data file 9). The transcriptomes of the immature siliques were omitted in this subtraction scheme because often the gametophyte enriched genes are also present in the developing embryo and endosperm. We found 129 gametophyte enriched and 108 embryo sac enriched genes, accounting for 10% and 8.6%, respectively, of the embryo sac expressed genes (Table 1). Among the embryo sac enriched genes, 52% are uncategorized, 17% are enzymes or genes that are involved in metabolism, 15% are involved in cell structure and transport, 8% are transcriptional regulators, 4% are involved in translational initiation and modification, 3% are predicted to be involved in RNA synthesis and modification, and 2% in signaling (Figure 1 and Table 1). Of the embryo sac enriched transcripts, 31% were present in the immature siliques, suggesting their expression in the embryo and endosperm (Table 1). Furthemore, 26% of the embryo sac enriched genes were similar to monocot ESTs from the embryo sac or egg, and 41% were similar to central cell and endosperm ESTs (Table 1).

**Table 1:** Enriched expression of genes in the embryo sac cells was distinguished by their absence of detectable expression in sporophytic and pollen transcriptomes

				Orthologous *Zm *EST^c^		
				
Gene ID	Gene description	Study^a^	Homology to *At *siliques transcriptome^b^	ES	Egg	CC and EN
Transcriptional Regulation						
At5g06070	Zinc Finger (C2H2 Type) Family Protein (RBE)	2	0	0	0	0
At1g75430	Homeodomain Protein	1	0	0	0	0
At2g01500	Homeodomain-Leucine Zipper (WOX6, PFA2)	1	0	0	0	1
At2g24840	MADS-Box Protein Type I (AGL61)	2	0	1	1	1
At1g02580	MEDEA (MEA)	2	0	0	1	1
At5g11050	MYB Transcription Factor (MYB64)	1, 2	0	0	0	1
At3g29020	MYB Transcription Factor (MYB110)	2	0	0	0	1
At5g35550	MYB Transcription Factor (MYB123) (TT2)	2	1	0	0	1
At4g18770	MYB Transcription Factor (MYB98)	2	0	0	0	1
Core Signaling Pathways						
At5g12380	Annexin	2	0	1	1	1
At2g20660	Rapid Alkalinization Factor (RALF)	2	0	0	0	0
RNA Synthesis And Modification						
At1g63070	PPR Repeat-Containing Protein	1	0	0	0	1
At2g20720	PPR Repeat-Containing Protein	1	0	0	0	0
At3g54490	RPB5 RNA Polymerase Subunit	2	0	1	1	1
Protein Synthesis And Modification						
At5g11360	Protein Involved in Amino Acid Phosphorylation	2	1	1	0	0
At4g15040	Subtilase Family Protein, Proteolysis	2	0	1	1	1
At5g58830	Subtilase Family Protein, Proteolysis	2	1	0	1	1
At1g36340	Ubiquitin-Conjugating Enzyme	2	1	1	1	1
Enzymes And Metabolism						
At3g30540	(1-4)-Beta-Mannan Endohydrolase Family	2	0	0	0	0
At1g47780	Acyl-Protein Thioesterase-Related	2	0	1	0	0
At1g31450	Aspartyl Protease Family Protein	2	0	1	1	1
At1g69100	Aspartyl Protease Family Protein	2	0	0	1	1
At2g28010	Aspartyl Protease Family Protein	2	0	0	1	1
At2g34890	CTP Synthase, UTP-Ammonia Ligase	2	1	1	0	1
At4g39650	Gamma-Glutamyltransferase	2	1	0	0	0
At4g30540	Glutamine Amidotransferase	2	0	0	0	1
At3g48950	Glycoside Hydrolase Family 28 Protein	2	1	0	1	1
At2g42930	Glycosyl Hydrolase Family 17 Protein	2	0	1	1	1
At4g09090	Glycosyl Hydrolase Family 17 Protein	2	1	1	1	1
At1g56530	Hydroxyproline-Rich Glycoprotein	1	0	0	0	0
At1g06020	Pfkb-Type Carbohydrate Kinase	2	1	0	1	1
At2g43860	Polygalacturonase	2	0	0	0	1
At1g78400	Glycoside Hydrolase Family 28 Protein	1	0	0	0	1
At5g22960	Serine Carboxypeptidase A10 Family Protein	1	0	0	1	1
At4g21630	Subtilase Family Protein	2	1	1	1	1
At4g26280	Sulfotransferase Family Protein	2	0	0	0	0
Cell Structure And Transport						
At1g10010	Amino Acid Permease Involved In Transport	2	1	1	0	0
At4g20800	FAD-Binding Domain-Containing Protein	2	0	0	1	0
At1g34575	FAD-Binding Domain-Containing Protein	1	1	0	1	0
At1g48010	Invertase/Pectin Methylesterase Inhibitor Family Protein	2	0	0	0	0
At3g17150	Invertase/Pectin Methylesterase Inhibitor Family Protein	2	1	0	0	0
At3g55680	Invertase/Pectin Methylesterase Inhibitor Family Protein	1	0	0	0	0
At2g47280	Pectinesterase Family Protein	2	0	0	0	0
At4g00190	Pectinesterase Family Protein	2	0	0	0	0
At5g18990	Pectinesterase Family Protein	2	0	0	0	0
At1g56620	Pectinesterase Inhibitor	2	0	0	0	0
At2g23990	Plastocyanin-Like	2	1	0	0	1
At1g73560	Lipid Transfer Protein (LTP) Family Protein	2	1	0	0	0
At5g56480	Lipid Transfer Protein (LTP) Family Protein	2	0	0	0	1
At1g63950	Lipid Transfer Protein (LTP) Family Protein	2	1	0	0	0
At3g05460	Sporozoite Surface Protein-Related	2	1	0	0	0
At5g06170	Sucrose Transporter	2	1	0	0	1
Uncategorized						
At1g24000	Bet V I Allergen Family Protein	2	1	0	0	0
At3g42130	Glycine-Rich Protein	1	0	0	0	0
At3g17140	Invertase Inhibitor-Related	2	0	0	0	0
At5g09360	Laccase-Like Protein Laccase	2	0	1	1	1
At1g79960	Ovate Protein-Related	2	0	0	0	0
At3g59260	Pirin	2	0	0	0	0
At4g30070	Plant Defensin-Fusion Protein	2	1	0	0	0
At5g38330	Plant Defensin-Fusion Protein	2	0	0	0	0
At2g01240	Reticulon Family Protein (RTNLB15)	2	0	1	0	0
At3g17080	Self-Incompatibility Protein-Related	2	0	0	0	0
At5g12060	Self-Incompatibility Protein-Related	2	1	0	0	0
At3g28020	Unknown	1	0	0	0	0
At3g19780	Unknown	1	0	0	0	0
At5g30520	Unknown	1	0	0	0	0
At3g45380	Unknown	1	0	0	0	0
At4g23780	Unknown	1	0	0	0	0
At1g54926	Unknown	1	0	0	0	0
At3g23720	Unknown	1	0	0	0	0
At1g47470	Unknown	1, 2	0	0	0	0
At1g32680	Unknown	1	0	0	0	0
At1g11690	Unknown	1	0	0	0	0
At2g04870	Unknown	1	0	0	0	0
At2g06630	Unknown	1	0	0	0	0
At4g09400	Unknown	1	0	0	0	0
At1g21950	Unknown	2	1	0	0	0
At4g11510	Unknown	2	1	0	0	0
At5g25960	Unknown	2	0	0	0	1
At1g60985	Unknown	2	0	0	0	0
At1g63960	Unknown	2	0	0	0	0
At1g78710	Unknown	2	1	1	0	1
At2g02515	Unknown	2	1	0	0	0
At2g20070	Unknown	2	0	0	0	0
At2g21740	Unknown	2	0	0	1	0
At2g30900	Unknown	2	0	1	0	1
At3g04540	Unknown	2	0	0	0	0
At3g13630	Unknown	2	1	0	0	0
At3g43500	Unknown	2	0	0	0	0
At3g57850	Unknown	2	0	0	0	0
At4g07515	Unknown	2	0	0	0	0
At4g10220	Unknown	2	1	0	0	0
At4g17505	Unknown	2	0	0	0	1
At5g17130	Unknown	2	0	0	0	0
At5g25950	Unknown	2	1	0	0	1
At5g46300	Unknown	2	1	0	0	0
At5g64720	Unknown	2	0	0	1	0
At1g52970	Unknown	2	0	0	0	0
At5g42955	Unknown	2	0	0	0	0
At2g21655	Unknown	2	0	0	0	0
At2g20595	Unknown	2	0	0	0	0
At1g45190	Unknown	2	0	0	0	0
At5g43510	Unknown	2	1	0	0	0
At2g15780	Unknown, Blue Copper-Binding Protein	1	0	0	0	0
At1g24851	Unknown	1	0	0	0	0
At1g30030	Non-LTR retrotransposon family (LINE)	1	0	ND	ND	ND
At2g34130	CACTA-like transposase family	2	0	ND	ND	ND
At3g42930	CACTA-like transposase family	1	0	ND	ND	ND

Embryo sac-enriched expression for the 1,260 candidate genes was deduced by comparing the transcriptomes of cotyledon, hypocotyls, root, leaf, shoot, petiole, sepal, petal, pedicel, mature siliques, mature seeds, rosettes, and pollen (see Additional data file 6 for details). Note that there were ten more microarray probes that identified expressed genes (At1g75610, At4g04300, At2g13750, At3g32917, At4g05600, At4g07780, At2g23500, At1g78350, At5g34990, and At2g10840), but they were omitted as pseudogenes by the The *Arabidopsis *Information Resource (TAIR) Gene ontology. See Additional data files 2 and 3 for further details. ^a^'1' indicates *coatlique *dataset and '2' indicates *sporocyteless *dataset. ^b^'0' indicates absent and '1' indicates present in *Arabidopsis thaliana *(*At*) transcriptomes of immature siliques with globular embryo. Data are derived from Schmid and coworkers [28]. ^c^Appropriate scores were assigned if an *Arabidopsis *gene is similar (= 1) or not (= 0) to *Zea mays *(*Zm*) and wheat expressed sequence tags (ESTs) by basic local alignment search tool (BLAST) analysis at an e-value of 10^-8^. A total of 10,747 *e*mbryo sac (ES) ESTs, 5,925 egg cell ESTs, and 15,677 ESTs from central cell (CC) and immature endosperm (EN) cells (1-6 days after pollination [DAP]) were used in the BLAST analysis. See Additional data file 8 for further details on the ESTs. ND, not determined.

### Targeted reverse genetic approaches identified female gametophytic and zygotic mutants

**Figure 3:**
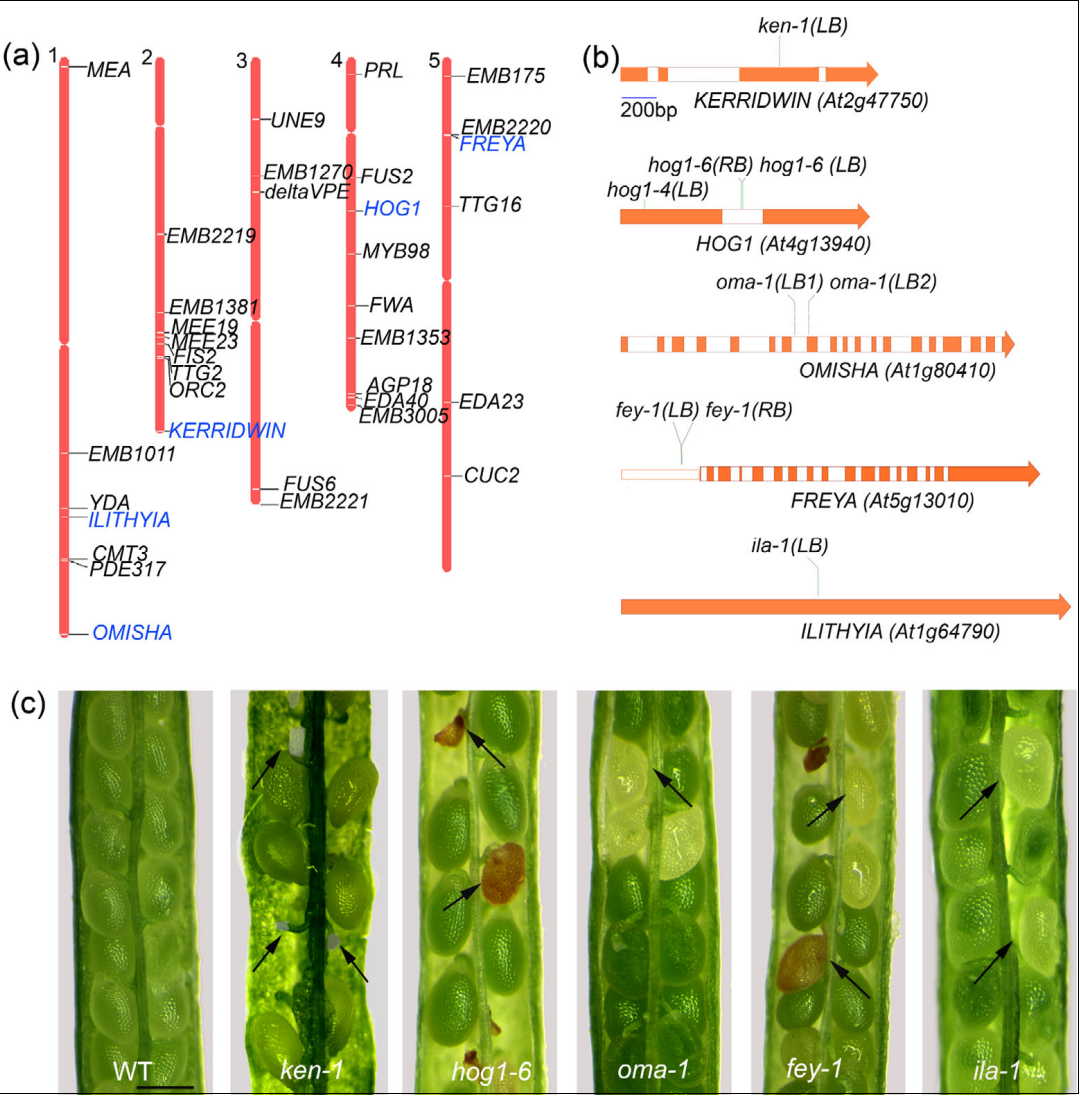
Genes essential for female gametogenesis, fertilization, and seed development are present in the embryo sac transcriptome datasets. **(a) **Chromosomal locations of 35 essential genes. Five genes that are described in the current work are shown in blue. Description of the mutants and corresponding references are given in Additional data file 5. **(b) **Five genes and the locations of corresponding mutant alleles described in this work. Exons are shaded in orange. The genes were named after the following Goddesses: *KERRIDWIN*, the Welsh triple Goddess of trinity known for nurturing children; *OMISHA*, Indian Goddess of birth and death; *FREYA*, the Norse Goddess of fertility; and *ILITHYIA*, the Greek Goddess of childbirth. *HOG1*, *HOMOLOGY DEPENDENT GENE SILENCING 1*; LB and RB, left and right borders of the T-DNA. **(c) **Mutants were identified based on infertile ovules (*ken-1*) or seed abortion (*hog1-6*, *oma-1*, *fey-1*, and *ila-1*). The arrows identify the defective ovules. Scale bar: 100 μm in panel c.

Initial examination of our dataset for previously characterized genes revealed that the dataset contained 33 genes that were reported to be essential for female gametophyte or seed development (Figure 3 and Additional data file 4). Given the availability of T-DNA mutants from the *Arabidopsis *stock centers, we wished to examine T-DNA knockout lines of some selected embryo sac expressed genes for ovule or seed abortion. During the first phase of our screen using 90 knockout lines, we identified eight semisterile mutants with about 50% infertile ovules indicating gametophytic lethality, and four mutants with about 25% seed abortion suggesting zygotic lethality (Table 2). When we examined the mutant ovules of gametophytic mutants, we found that seven mutants exhibited a very similar terminal phenotype: an arrested one-nucleate embryo sac. Co-segregation analysis by phenotyping and genotyping of one such mutant, namely *frigg *(*fig-1*) demonstrated that the mutant was not tagged, and the phenotype caused by a possible reciprocal translocation that may have arisen during T-DNA mutagenesis (Table 2). Preliminary data suggested that the six other mutants with a similar phenotype were not linked to the gene disruption either. Although not conclusively shown, it is likely that these mutants carry a similar translocation and, therefore, we did not analyze them further. These findings demonstrate that among the T-DNA insertation lines available, a rather high percentage (7/90 [8%]) exhibit a semisterile phenotype that is not due to the insertion. Therefore, caution must be exercised in screens for gametophytic mutants among these lines.

**Table 2:** Genetics of mutant alleles affecting the female gametophyte and seed development

Mutant^a,b^	Segregation ratio^c,d^	χ^2 ^(segregation ratio)^e^	Seed abortion^f^	χ^2 ^(seed abortion)^g^	Mutant embryo sac phenotype
*ken-1*	0.97 (*n *= 290)	0.06**	54% (*n *= 327)	2.23*	54% unfused polar nuclei (*n *= 327)
*fig-1*^h^	ND	ND	53% (*n *= 258)	0.99*	53% arrested one-nucleate embryo sac (*n *= 258)
*hog1-4*	1.96 (*n *= 548)	0.04**	26% (*n *= 552)	0.24*	ND
*hog1-6*	2.11 (*n *= 351)	0.21**	24% (*n *= 420)	0.11*	22% aberrant early endosperm mitosis and zygote (*n *= 318)
*oma-1*	2.10 (*n *= 251)	0.13**	18% (*n *= 514)	13.1^#^	17% arrested, arrested mid-globular embryo (*n *= 269)
*fey-1*	1.99 (*n *= 425)	0.00**	21% (*n *= 414)	4.41**	19% arrested, arrested late-globular embryo (*n *= 243)
*ila-1*	1.92 (*n *= 038)	0.01**	23% (*n *= 352)	0.74*	20% and 3% arrested torpedo and late heart embryo (*n *= 352)

^a^The stock IDs of the mutant alleles are as follows: SM_3_23805, SALK_000711, CSHL_GT1724, Syngenta_18372 (EMB1395), GENOPLANTE_FBV_6 (EMB3011), Syngenta_102828 (EMB2753) and SALK_119854. ^b^*hog1-4 *is in L*er* background, *hog1-6 *and *fey-1 *in *Ws*, and the other four mutants in *Col *background. ^c^Segregation ratio was calculated as a ratio of resistant to sensitive plants upon appropriate progeny selection in antibiotic on Murashigge and Skoog medium (*n *= total number of progeny). ^d^*ken-1*, *hog1-6*, and *oma-1 *were resistant to glufosinate-ammonium; *hog1-4 *was kanamycin resistant; PCR genotyping was done for *fig-1 *and *ila-1 *where the kanamycin selection was not possible due to gene silencing; *ken-1 *and *hog1-6 *exhibited partial silencing of the selection marker in later generations. ^e^χ^2 ^statistic was calculated with the segregation ratio expectation of 1:1 for female gametophytic mutants and 2:1 for the zygotic mutants. Probability (*P*) values for the χ^2 ^values are as follows: *P = 0.05, **P = 0.01, and ^#^*P *= 0.0004. ^f^In *ken-1 *and *fig-1 *the ovules were infertile, and they arrested before seed development. ^g^χ^2 ^statistic for seed abortion was calculated with the aborted-to-normal expectation of 1:1 for female gametophytic mutants (*ken-1 *and *fig-1*) and 1:3 for the zygotic mutants (*hog1-4*, *hog1-6*, *fey-1*, *oma-1*, and *ila-1*). ^h^Left-border of the T-DNA was confirmed to be inserted in the first intron of at4g30840; the genotype did not co-segregate with the semi-sterile phenotype; similar phenotypic data were obtained for seven mutants in other genes and are thought to be unrelated to the insertions (data not shown). ND, not determined.

**Figure 4:**
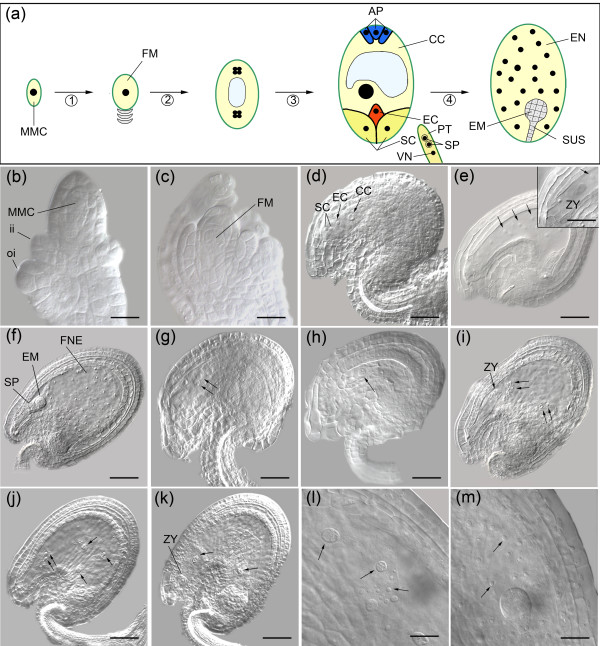
Female gametophytic and early zygotic mutant phenotypes highlight the essential role of corresponding genes for reproductive development. **(a) **A cartoon showing the ontogeny of the wild-type female gametophyte in *Arabidopsis *and the early transition to seed development. A haploid functional megaspore (FM) develops from a diploid megaspore mother cell (MMC) upon two meiotic divisions (1). Three syncitial mitotic divisions (2) convert the FM into an eight-nuclear cell. Upon nuclear migration, cellularization, nuclear fusion and differentiation (3), a cellularized seven-celled embryo sac forms. It contains an egg cell (EC) and two synergid cells (SC) at the micropylar pole, three antipodals (AP) at the chalazal pole, and one vacuolated homo-diploid central cell (CC) in the middle. Subsequently, the AP cells degenerate. Degeneration of one SC precedes the entry of one pollen tube (PT), and two sperm cells (SP) independently fertilize the egg and central cell, leading to the development of a diploid embryo (EM) and triploid endosperm (EN) respectively. SUS, suspensor, VN, vegetative nucleus. **(b-f) **Morphology of wild-type ovules corresponding to representative events described above is depicted (ii indicates inner integuments, and oi indicates outer integuments). Both synchronous and asynchronous free nuclear mitotic divisions (as shown in panel e; arrows) lead to development of the free nuclear endosperm (FNE) as shown in panel f. The insert in panel e depicts a developing zygote (ZY). **(g) **In *kerridwin *(*ken-1*), two polar nuclei in the central cell fail to fuse. **(h) **Female gametophyte development did not initiate beyond the one-nucleate embryo sac stage (arrows) in *frigg *(*fig-1*). **(i-l) **Anomalies in early endosperm and zygotic development in *hog1 *(*homology dependent gene silencing 1*) mutants. The zygote did not develop beyond single cell stage, and subsequent divisions and cytokinesis did not occur (panel i, j, and k). The arrows in panels i and j identify the irregular nature of free nuclear mitotic divisions in *hog-1 *endosperm. The endosperm nuclei were irregular in size and they were often clustered. Compare the large and small irregular endosperm nuclei in *hog1-6 *(panel l) with the regular free nuclear endosperm nuclei in **(m) **the wild type. Scale bars: 20 μm for panels d to k, and the insert of panel e; and 50 μm in panels b, c, l, and m.

In about 54% of the ovules, the polar nuclei failed to fuse in *kerridwin *(*ken-1*), a mutant allele of *AT2G47750*, which encodes an auxin-responsive GH3 family protein (Figure 4 and Table 2). The corresponding wild-type pistils exhibited 9% unfused polar nuclei when examined 2 days after emasculation, and the remaining ovules had one fused central cell nucleus (*n *= 275). The *hog1-6 *mutant is allelic to the recently reported *hog1-4*, disrupting the *HOMOLOGY DEPENDENT GENE SILENCING 1 *gene (*HOG1*; *AT4G13940*), and they both were zygotic lethal, producing 24% to 26% aborted seeds (Table 2) [55]. Both these mutants exhibit anomalies during early endosperm division and zygote development (Figure 4i-l). In wild-type seeds, the endosperm remains in a free-nuclear state before cellularization around 48 to 60 hours after fertilization (HAP), and the embryo is at the globular stage (Figure 4f). In *hog1-6*, at about the same time the endosperm nuclei displayed irregularities in size, shape and number, and they never were uniformly spread throughout the seed (Figure 4i-l; *n *= 318). The irregular mitotic nuclei were clustered into two to four domains. The zygote remained at the single-cell stage, and in 2% of the cases it went on to the two-cell stage. In very rare instances (five observations), two large endosperm nuclei were observed while the embryo remained arrested at single-cell stage in *hog1-4 *(Figure 4k).

**Figure 5:**
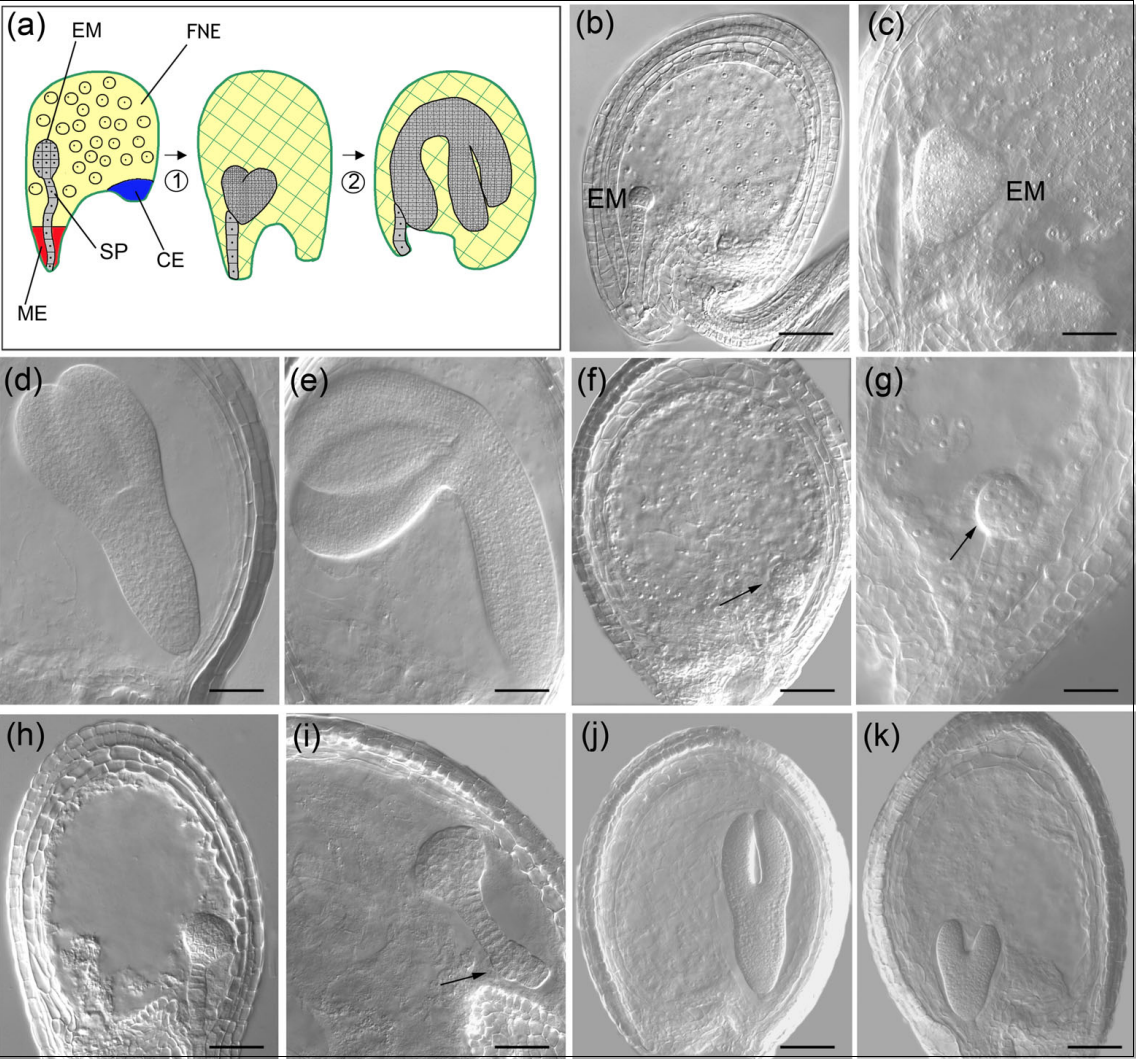
Mutants arrested late in seed development. **(a) **Shown is a scheme of seed development in *Arabidopsis*. A globular embryo (EM) develops into heart stage (1). Note that the peripheral endosperm nuclei surrounding the globular embryo are organized into three distinct domains: micropylar endosperm (ME), chalazal endosperm (CE), and free nuclear endosperm (FNE). Following rapid cellularization of endosperm, a torpedo stage embryo and then an upturned-U stage embryo is formed (2). **(b-e) **Morphology of wild-type seed development corresponding to representative events described above. **(f) **In *oma-1 *the embryo arrested at the mid-globular stage. The size of cells in embryo and endosperm were larger than that in **(g) **the wild type. **(h,i) **In *fey-1 *the embryo arrested at around the late globular stage. Note that the cells of the embryo and suspensor were large, and the suspensor displays a bend due to the irregularly bulged cells (panel i, arrow). **(j) **The majority of the *ila-1 *embryos arrested when they were at upturned U stage. **(k) **A small fraction of late-heart *ila-1 *embryos could also be observed. Scale bars: 10 μm for panels b, f, h, j, and k; and 20 μm for panels c, d, e, g, and i.

In *omisha *(*oma-1*) and *freya *(*fey-1*), the T-DNA disrupted *AT1G80410 *(encoding an acetyl-transferase) and *AT5G13010 *(encoding an RNA helicase), leading to 18% and 21% seed abortion, respectively (Table 2). The embryo arrested around the globular stage in both mutants (Figure 5f-i). The arrested mid-globular embryo cells (17%; *n *= 269) were larger in size in *oma-1*, whereas the corresponding wild type progressed to late-heart and torpedo stages with cellularized endosperm (Figure 5g). In the aborted *fey-1 *seeds, the cells of late-globular embryos (19%; *n *= 243) were much larger and irregular in shape than in the wild type, but no endosperm phenotype was discernible (Figure 5i). In most cases, giant suspensor cells were seen in *fey-1*, and there were more cells in the mutant suspensor than in that of the wild type (Figure 5i). *ILITHYIA *disrupts *AT1G64790 *encoding a translational activator, and the *ila-1 *embryos arrested when they reached the torpedo stage (Figure 4j and Table 2; *n *= 352). A small proportion of *ila-1 *embryos arrested at a late heart stage (11 observations). The results from the first phase of our targeted reverse genetic approach showed that there are mutant phenotypes for embryo sac expressed candidate genes, and that these gene disruptions lead to lethality during female gametophyte or seed development.

### Transcription factors, homeotic genes, and signaling proteins are over-expressed in the absence of an embryo sac

Even though the two mutants we used in this study exhibit morphologically normal carpels and ovules in the absence of an embryo sac, we considered whether the gene expression program within the sporophyte is altered. The genes exhibiting higher levels of expression in the *coa *and *spl *mutants could be regarded as candidate genes that were deregulated in the maternal sporophyte because of the absence of a functional embryo sac in these mutants. Of the 527 genes identified for their maternal-gain-of-expression in *coa *and *spl*, about 9% were predicted to be involved in transcriptional regulation and 7% were signaling proteins (Figure 1). Among the genes encoding transcription factors, there were eight MYB class protein genes, seven zinc-finger protein genes including *SUPERMAN *and *NUBBIN*, five homeo box genes including *SHOOT MERISTEMLESS *(*STM*), five genes each encoding basic helix-loop-helix (bHLH) and SQUAMOSA-binding proteins, three genes encoding basic leucin zipper (bZIP) proteins, and two genes each encoding APETALA2-domain and NAC-domain transcription factors. No MADS box genes were represented. The genes encoding signaling proteins included the auxin-responsive genes *AUXIN RESISTANT 2/3 *(*AXR2 *and *AXR3*), three genes encoding DC1-domain-containing proteins, ten genes encoding kinases and related proteins, two genes encoding phosphatases, four LRR-protein genes, five auxin response regulator genes, and the two zinc-finger protein genes *SHORT INTERNODES *(*SHI*) and *STYLISH2 *(*STY2*; Additional data file 5). When we examined the whole dataset for genes encoding secreted proteins, 87 predicted proteins fulfilled the criteria; 24% were below 20 kDa in size, which included a peptidase and two lipid transfer proteins (data not shown).

### The carpel is the major target tissue for over-expression caused by the lack of an embryo sac

**Figure 6:**
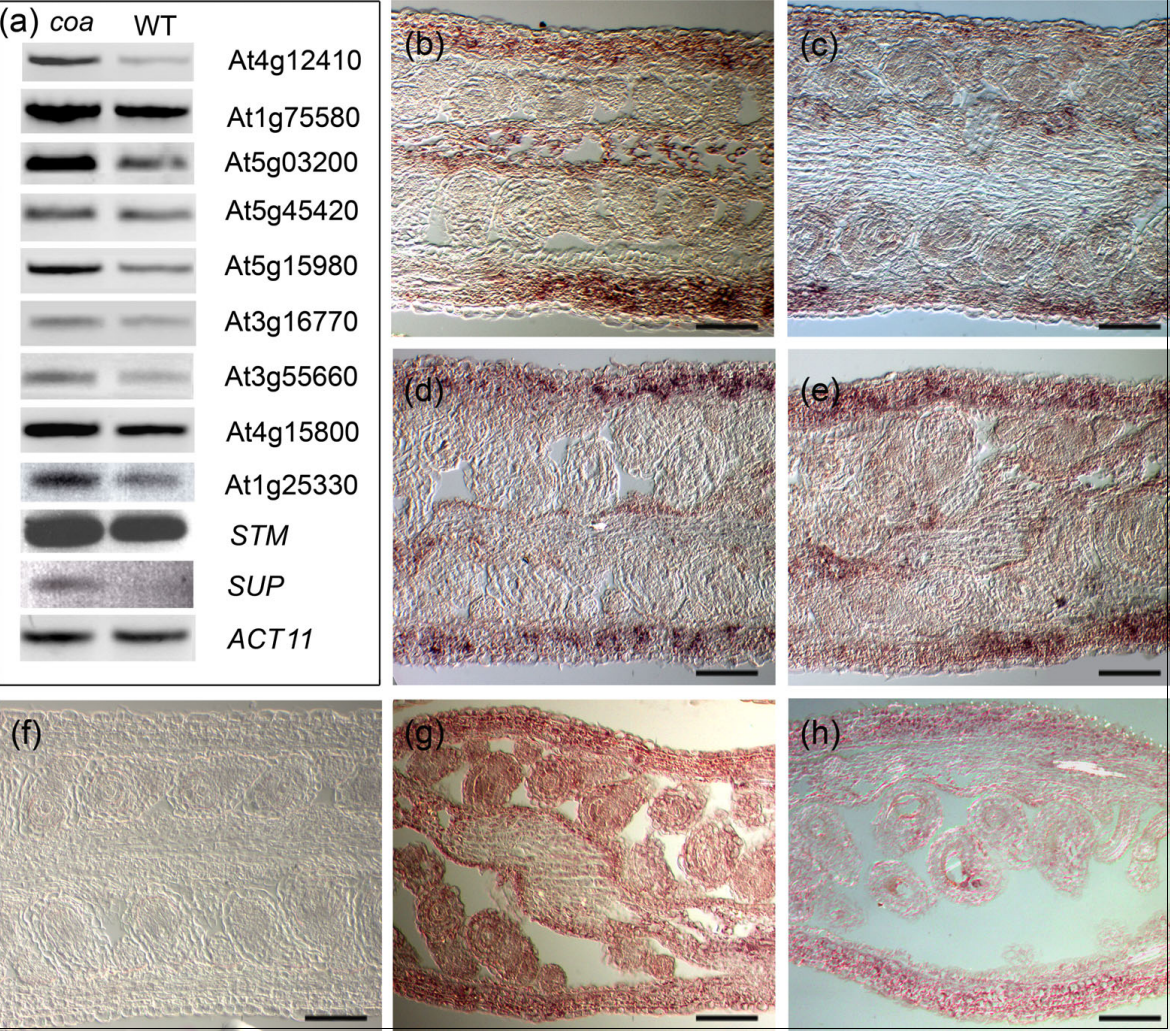
Gain of expression in the sporophyte in the absence of a functional embryo sac: expression analysis in the *coatlique *(*coa*) mutant. **(a) **RT-PCR for 11 genes in *coa *and wild-type (WT) pistils. Equal loading of both *coa *and WT cDNA templates in PCR was monitored by expression of *ACT11*. *SUP*, *SUPERMAN*. Also shown are *in situ *expression patterns of the following genes in *coa *pistil tissues: **(b) ***AT4G12410*, encoding an auxin-responsive Small Auxin Up RNA (SAUR) protein; **(c) ***AT1G75580*, encoding an auxin-responsive protein; **(d) ***AT5G03200*, encoding a C3HC4-type RING finger protein; and **(e) **at5g15980, encoding a PPR repeat containing protein. The corresponding sense control probes did not show any expression (data not shown). **(f) ***AT4G12410 *did not show any detectable expression pattern in wild-type pistils. The other four genes exhibited spatial expression patterns in the wild-type ovule and carpel tissues comparable to that of *coa*, but their wild-type expression levels were much lower than in *coa *(data not shown). **(g) **We initially identified the over-expression of *STM *in the ovule tissues of *spl *(*sensu *microarray data), and confirmed that this gene is over-expressed in the carpel and ovules of *coa *as well (panels a and g). **(h) **A comparable but less intense spatial expression pattern of *STM *was seen in wild-type pistils. Scale bars: 100 μm in panels b to h.

In order to confirm that the genes we identified truly reflect a gain of expression in the maternal sporophyte of the mutant, we examined the expression levels and patterns of 11 candidate genes in *coa *and wild-type gynoecium by RT-PCR or *in situ *hybridization. Figure 6a shows an RT-PCR panel confirming that eight genes from the *coa *dataset and three genes from the *spl *dataset were more highly expressed in *coa *than in wild-type pistils. We present evidence that the genes we identified for their gain of expression in *spl *were indeed over-expressed in *coa *as well, suggesting that the genes are generally over-expressed in the absence of an embryo sac, regardless of the mutation (Figure 6a). Figure 6 shows the expression of the following genes in the *coa *gynoecium as detected by *in situ *hybridization: *AT4G12410 *(a *SAUR *[auxin-responsive Small Auxin Up RNA] gene; Figure 6b), *AT1G75580 *(an auxin-responsive gene; Figure 6c), *AT5G03200 *(encoding C3HC4-type RING finger protein; Figure 6d), *AT5G15980 *(encoding PPR repeat-containing protein; Figure 6e), and *STM *(a homeo box gene; Figure 6g). Surprisingly, all of the five genes exhibited similar expression patterns: strong expression in the carpel wall and septum, and relatively low expression in the sporophytic tissues of the ovules surrounding the embryo sac. In case of *AT4G12410*, we did not detect expression in the wild-type pistils. For the other four genes, the spatial expression patterns in the wild-type ovule and carpel tissues were comparable to that in *coa*, but the expression levels were far lower than in the mutant (data not shown). In summary, we provide evidence that a significant fraction of the sporophytic transcriptome can be modulated by the presence or absence of an embryo sac.

## Discussion

### A comparative genetic subtraction approach identifies embryo sac expressed candidate genes

The female gametophyte or the embryo sac develops from a single functional megaspore cell through a series of highly choreographed free-nuclear mitotic divisions [1,2]. Understanding the molecular pathways that govern embryo sac development and function, as well as subsequent seed development, has important implications for both basic plant developmental biology and plant breeding. Despite the possible involvement of a few thousands of genes in this essential developmental pathway, only a few more than 100 genes have been identified by loss-of-function mutations, and most of them have not been studied in detail [14]. In the present study we provide an alternative strategy to identify genes that are expressed in the embryo sac of *A*. *thaliana*, namely comparative whole-genome transcriptional profiling by microarray, which led to a candidate dataset of 1,260 genes.

Our approach, similar to that employed by Yu and coworkers [34], is different from that used in previously reported whole-genome transcriptional profiling experiments (for example, pollen transcriptome [33] and whole flower and silique transcriptome [29]) in that we deduced the transcriptome of the few-celled female gametophyte by simple genetic subtraction using a mutant that lacks an embryo sac. Putative embryo sac expressed candidates included a significant number of genes that are involved in transcriptional regulation, signaling, translational regulation, protein degradation, transport and metabolism, and a majority of genes that were not identified in previous studies. Similar to previous transcriptional profiling reports, the largest functional category of embryo sac expressed genes was plant metabolism [29,52,56]. Percentages of genes classified into transcriptional regulation and signaling were comparable across embryo sac and pollen expressed transcriptomes (about 6% to 10%) and, interestingly, these categories are larger in both gametophytic transcriptomes than the general sporophytic transcriptomes such as leaf, stem, and root [28]. In a much larger dataset of pollen samples, Pina and colleagues [33] reported a little over 16% of pollen expressed genes as part of the signaling category. It is possible that the mature pollen transcriptome is more active in terms of signal transduction processes than that of the embryo sac, given its role during polarized tip growth through the female reproductive tract, and the gametic interaction at fertilization (for review [57]). We could not compare other functional categories across other organ-specific transcriptome datasets because the methods employed for functional classification were very different. Briefly, our work provides novel data for organ specific expression in *Arabidopsis *and, in particular, it illustrates the similarities and dissimilarities between male and female gametophytic expression.

Interesting insights can be gained from the subset of embryo sac expressed genes (8.6%) that was subtracted for their enriched expression only in the embryo sac. It was recently reported that 10% to 11% of the pollen transcriptome was selectively expressed in the pollen, as evident from their absence of expression in the sporophytic transcriptomes (*n *= 1,584 in [30] and *n *= 6,587 in [33]). In a very similar study [32], it was reported that 9.7% of the 13,977 male gametophytically expressed genes were specific for the male gametophyte. Even though the complete embryo sac transcriptome is yet to be determined, it appears that the enriched transcriptome of the embryo sac we report here is similar in size to that of pollen. Male and female gametophyte enriched transcriptomes appear to be much larger than the specific transcriptomes of vegetative organs such as leaf and entire seedlings, which accounted for 2% to 4% of their corresponding complete transcriptomes [33]. When we compared the genes with enriched expression in the embryo sac or pollen, the embryo sac appears to harbor more transcriptional regulators than pollen (8% versus 3%) [30]. However, the pollen transcriptome exhibited a greater abundance of signaling proteins than the embryo sac (23% versus 2%). This implies that either the pollen is more active in signaling than the female gametophyte at the time around fertilization, or that the sensitivity of detecting signaling genes in the embryo sac will have to be improved in the future studies. The promise of our approach to deducing genes with enriched expression was supported by the presence of essential genes that are female gametophyte specific, such as *MEDEA *and *MYB98 *in our dataset [12,22]. Furthermore, temporal and spatial expression of nine transcripts in this study, and 18 other genes from previous studies, suggests that the whole dataset of embryo sac expressed genes may comprise genes that are expressed either in the entire embryo sac or restricted to a few or single cell types (Additional data file 1 and the references therein).

A significant fraction of genes were probably undetected by this experiment for two reasons: relatively similar or higher expression in the maternal sporophytic tissues; and low level of expression in the embryo sac, similar to most of the known female gametophytic genes. For example, cell cycle genes are barely represented among our candidate genes. In contrast, the pollen transcriptome has been reported to be enriched with several core cell cycle transcripts [33]. Although our comparative approach is very different from that reported by Pina and coworkers [33], there could be a large number of cell cycle regulators that are expected to be expressed during embryo sac development, suggesting a need for improvements in embryo sac isolation and subsequent transcriptome analysis. Unlike the relative ease in isolating some embryo sac cell types in maize and wheat, large-scale isolation of the embryo sac cells is not possible in *Arabidopsis *[58,59]. Following the work conducted by Yu and coworkers [34], we present here a large-scale study to explore embryo sac expressed genes in *Arabidopsis*. If the scale of gene discovery is to be improved much further, then methods to isolate embryo sac cells using methods such as florescence-activated cell sorting, targeted genetic ablation by expression of a cell-autonomous cytotoxin, or laser-assisted microdissection must be developed [51,60-62].

### The embryo sac expressed candidate genes may be essential for female gametophyte and seed development

Once we had validated the expression of the embryo sac expressed genes, we considered whether these genes could play essential roles during embryo sac and seed development. It is apparent from our work on five mutants, and mutant data from the literature, that the embryo sac expressed genes that we report here may play a crucial role during the embryo sac development or later during seed formation. *HOG1 *is of special interest because we have provided evidence for allelic phenotypic complementation by two mutant alleles. *HOG1 *is proposed to act upstream of *METHYL TRANSFERASE 1 *(*MET1*) and *CMT3 *among other methylases, and mutants for *HOG1 *have high levels of global hypomethylation [54]. It has become clear that DNA hypomethylation plays a crucial role during gametogenesis, and that mutations affecting the genes in this pathway such as *HOG1*, *MET1*, and *CMT3 *affect embryo and endosperm development [55,63,64]. It is interesting to note that we identified *CMT3*, *MEA*, and *FIS2 *that are associated with pathways involving DNA and histone methylation [63,65-68].

We have shown that our dataset will be a resource for targeted reverse genetic approaches. The extensive reverse genetic tools available for *Arabidopsis *researchers make such a large-scale functional study possible [69]. While screening for female gametophytic mutants through T-DNA mutagenesis, we unexpectedly observed a number of female gametophytic mutants that had a very similar phenotype: a complete arrest of female gametogenesis at the one-nuclear stage. These, however, were not linked to the gene disruption. *Agrobacterium*-mediated *Arabidopsis *T-DNA mutagenesis has been facilitated by floral dipping, which involves integration of the T-DNA through the ovule, and the chromosomes of the female gametophyte are the main target for T-DNA insertion [70]. Based on our results from this study, and other independent observations (Johnston AJ, Grossniklaus U, unpublished data), we believe that these unlinked gametophytic lethal events arose because of translocations and other rearrangements of maternal chromosomes during the integration of the T-DNA, and we advise due caution in mutant screening.

### Communication between the embryo sac and the surrounding sporophyte may be important for reproductive development

In *Arabidopsis*, the sporophytic and gametophytic tissues are intimately positioned next to each other within the ovule. Independent studies on *Arabidopsis *ovule mutants suggest that the development of the female gametophyte might require highly synchronized morphogenesis of the maternal sporophyte surrounding the gametophyte [1,35,37]. This notion is exemplified by the fact that megagametogenesis is largely perturbed in most of the known sporophytic ovule development mutants. For example, in *short integument 1 *(*sin1*) the ovules display uncoordinated growth patterns of integuments and the nucellus, and embryo sac development is not initiated [35,71]. In *bell1 *and *aintegumenta *mutants, in which integument morphogenesis and identity are disrupted, embryo sac development is arrested [35,37,72,73]. Therefore, early acting sporophytic genes in the ovule also affect female gametophyte development. On the contrary, in several mutations where female gametogenesis is completely or partially blocked, the ovule sporophyte appears morphologically normal. In *coa *and *spl*, or female gametophytic mutations such as *hadad *and *nomega*, embryo sac development is blocked either at the onset or during megagametogenesis, but ovule morphogenesis continues normally until anthesis [8,18,38]. It was therefore thought that the embryo sac does not influence the development of the sporophytic parts of the surrounding ovule and carpel tissues [2].

Our data clearly demonstrate that in the absence of an embryo sac there was a predominant transcriptional upregulation of transcription factors, and signaling molecules in the carpel and the ovule. It is interesting to note that we identified genes that were previously implicated in gynoecium patterning such as *NUBBIN*, *SHI *and *STY2 *for their gain of expression in the sporophyte [74-77]. Based on the proposed functionalities of these and other genes in our dataset, we suggest that signaling pathways involving auxin and gibberellic acid could possibly be triggered in the carpel and ovule sporophyte, in the absence of an embryo sac. We anticipate that sporophytic patterning genes and signaling molecules are under indirect repressive control by the female gametophyte. Impairment of this signaling cascade leads to deregulation of the sporophytic transcriptome.

## Conclusion

Understanding gene expression and regulation during embryo sac development demands large-scale experimental strategies that subtract the miniature haploid embryo sac cells from the thousands of surrounding sporophytic cells. We used a simple genetic subtraction strategy, which successfully identified a large number of candidate genes that are expressed in the cell types of the embryo sac. The wealth of data reported here lays the foundation to elucidate the regulatory networks of transcriptional regulation, signaling, transport, and metabolism that operate in these unique cell types of the haploid phase of the life cycle. Given that many of the genes in our expression dataset are essential to female gametophyte and seed development, targeted functional studies with further candidate genes promise to yield novel insights into the development and function of the embryo sac. Another major finding of this work is the identification of 108 genes that are enriched for embryo sac expression and thus probably play important roles for the differentiation and function of these specific cell types. The surprising finding that many genes are deregulated in sporophytic tissues in the absence of an embryo sac suggests a much more complex interplay of the haploid gametophytic with the diploid sporophytic tissues than was previously anticipated. Understanding the sporophytic regulatory network governed by the embryo sac will be of key interest for future studies.

## Materials and methods

### Plant material and growth conditions

The *coatlique *(*coa*) mutant was identified in *Arabidopsis *var Landsberg (*erecta *mutant; L*er*) background and L*er *was used as a wild-type control in the microarray and *in situ *hybridization experiments. Before transplanting, seeds were sown on Murashige and Skoog media (1% sucrose and 0.9% agar; pH 5.7) supplemented with appropriate selection markers and stratified for two days at 4°C (see Table 1 for description of mutants plants and selection markers). The seeds were germinated and grown for up to 15 days under 16-hour light/8-hour dark cycles at 22°C. Plants were then transplanted into ED73 soil (Einheitserde, Schopfheim, Germany) and grown in greenhouse conditions under a 16-hour photo-period at 22°C and 60% to 70% relative humidity.

### Histological analysis

For phenotypic characterization, the gynoecia of *Arabidopsis *wild-type, *coa *and gametophytic mutants, and siliques of the zygotic mutants were cleared in accordance with a protocol described in the report by Yadegari and coworkers [78]. Samples were observed using a Leica DMR microscope (Leica Microsystems, Mannheim, Germany) under differential interference contrast (DIC) optics.

### Transcriptional profiling by oligonucleotide array

Transcriptional profiling by Affymetrix microarray using *coa *and wild-type pistils, and downstream data analysis of the embryo sac and sporophytic transcriptomes are described in detail in Additional data file 10. In particular, emphasis was given to the low-level analysis of the microarray data, because the low fold change cut-off used for the embryo sac dataset could potentially introduce a large number of false positives. We chose to use three independent statistical packages (dCHIP, gcRMA and Gene Spring), with the most and least stringent being dCHIP and Gene Spring analysis, respectively. For dCHIP analysis, only those genes within replicate arrays called 'present' within a variation of 0 < median (standard deviation/mean) < 0.5 were retained for downstream analysis. By setting *P *to < 0.1 and differential fold change expression cut-off to 1.28-fold, we could predict that the median FDR ranges from 1% (*spl *dataset) to 3% (*coa *dataset) in the dCHIP analysis. The dilution of gametophytic cells in an excess of sporophytic tissues was higher in *coa *samples than in *spl *samples (discussed in Results, above), which may be the reason for the increase in the FDR. In such cases, standard error values of the signal averages, as given in the Additional data files 2 and 3, provide an indication for manual omission of false positives. In the analysis using gcRMA, pre-processed signal values were statistically analyzed using an empirical bayesian approach (see Additional data file 10) and the FDR was calculated for each gene using the options implemented in the Bioconductor software version 2.3.0 [79]. Only those genes with a FDR below 0.05 were considered to be differentially expressed. Manual omission of false-positive findings is possible in this type of analysis, if the standard error estimates of the mean RMA values (signal) and the absolute FDR values are to be used as indicators of false discovery. The sporophytic datasets did not impose such problems because the fold change cut-off was set to twofold as a stringent baseline, in addition to the analysis using three statistical methods.

### Bioinformatics analyses

The candidate genes were functionally classified according to the Gene Ontology data from TAIR or published evidence where appropriate. Annotations were improved mainly for the transcription factors from the *Arabidopsis *Gene Regulatory Information Server [80]. The secreted proteins were chosen based on the protein sequence analysis using TargetP with the top two reliability scores out of five [81]. A total of 32,349 maize and wheat EST sequences extracted from libraries specific for the embryo sac, egg, central cell, and early endosperm were obtained from various sources (see Additional data file 8 for details). The pools of EST sequences were converted to local BLASTable databases using NCBI software [82]. A PERL script was written to perform the mapping of *A. thaliana *female gametophyte transcriptome data to the EST datasets. An EST sequence is considered similar to an *Arabidopsis *protein if it matches at an e-value cutoff threshold of 10^-8 ^by TBLASTN [81]. For comparisons with sporophytic transcriptomes, the highly standardized experiment conducted by Schmid and coworkers [28] was chosen. Presence/absence calls calculated from the microarray analyses were downloaded for selected tissues from the TAIR website [83]. A gene was declared to be expressed in a tissue when a presence call was assigned to it in at least two out of three replicates. Details of the tissue-specific transcriptomes used are given in Additional data file 9.

### *In situ *hybridization

Inflorescences and emasculated pistils were paraplast embedded using the protocol of Kerk and colleagues [84] with minor modifications. Unique gene-specific probes of about 200 to 300 base pairs were cloned into pDRIVE (Qiagen, Basel, Switzerland) and used as templates for generating digoxygenin-UTP-labeled riboprobes by run-off transcription using T7 RNA polymerase, in accordance with the manufacturer's protocol (Roche Diagnostics, Basel, Switzerland). *In situ *hybridization was performed on 8 to 10 μm semi-thin paraffin sections, as described by Vielle-Calzada and coworkers [85] with minor modifications.

### Histochemical GUS expression

Embryo sac expression of the GUS reporter gene (β-glucuronidase) in the promoter-GUS lines and transposants was detected as described by Vielle-Calzada and coworkers [86].

### PCR primers and conditions

The sequences of all of the primers used in genotyping, RT-PCR, and *in situ *probe preparation, and the appropriate PCR conditions, are presented in Additional data file 11.

### Image processing

All of the images were recorded using a digital Magnafire camera (Optronics, Goleta, CA, USA), and they were edited for picture quality using Adobe Photoshop version CS (Adobe Systems Inc., San Jose, CA, USA).

## Abbreviations

BLAST, basic local alignment search tool; *coa*, *coatlique *mutant; dCHIP, DNA-Chip Analyzer; FDR, false discovery rate; gcRMA, GC robust multi-array average; MAS, MicroArray Suite (Affymetrix); RT-PCR, reverse transcription polymerase chain reaction; *spl*, *sporocyteless *mutant; TAIR, The *Arabidopsis *Information Resource.

## Authors' contributions

UG conceived of and supervised the project. AJJ, PM and UG designed and interpreted the experiments. AJJ, PM, JG and MF performed the experiments. AJJ, SEJW, ES and JDB contributed statistical and bioinformatics analyses. UG contributed reagents and materials. AJJ and UG wrote the paper.

## Additional data files

The following additional data are available with the online version of this paper. Additional data file 1 is a table listing the gene validation for embryo sac expression. Additional data file 2 lists the identity of embryo sac expressed genes, as revealed by genetic subtraction of *coa *from the wild type. Additional data file 3 lists the embryo sac expressed genes, identified by a reanalysis of the previously published dataset using the *spl *mutant [34]. Additional data file 4 lists genes from this work that were previously identified as being essential for reproductive development. Additional data file 5 lists those genes that were found to be over-expressed in the carpel and ovule tissues of *coa *and *spl *in the absence of an embryo sac. Additional data file 6 illustrates the scale of gene discovery by three independent methods across two types of datasets from two mutants. Additional data file 7 tabulates gene identities and the statistical treatments, confirming the necessity of different statistical treatments to identify embryo sac-expressed genes. Additional data file 8 lists the identifiers of maize and wheat ESTs from the embryo sac cell types, which were used in BLAST analysis of *Arabidopsis *proteins. Additional data file 9 provides details of previously reported transcriptome datasets used in data comparison. Additional data file 10 describes the methodology employed for transcriptional profiling by oligonucleotide array. Additional data file 11 lists the primers used for mutant genotyping, probes for mRNA *in situ *hybridization and RT-PCR.

The microarray CEL files used in this study are available from the Array Express (E-MEXP-1246).

## Supplementary Material

Additional data file 1 Presented is a table listing the gene validation for embryo sac expression. Click here for file

Additional data file 2 Presented is a list of the identity of embryo sac expressed genes, as revealed by genetic subtraction of *coa *from the wild type. Click here for file

Additional data file 3 Presented is a list of embryo sac expressed genes, identified by a reanalysis of the previously published dataset using the *spl *mutant [34]. Click here for file

Additional data file 4 Presented is a list of genes from this work that were previously identified as being essential for reproductive development. Click here for file

Additional data file 5 Presented is a list of those genes that were found to be over-expressed in the carpel and ovule tissues of *coa *and *spl *in the absence of an embryo sac. Click here for file

Additional data file 6 Illustrated is the scale of gene discovery by three independent methods across two types of datasets from two mutants. Click here for file

Additional data file 7 Presented is a table summarizing gene identities and the statistical treatments, confirming the necessity of different statistical treatments to identify embryo sac expressed genes. Click here for file

Additional data file 8 Listed are the identifiers of maize and wheat ESTs from the embryo sac cell types, which were used in BLAST analysis of *Arabidopsis *proteins. Click here for file

Additional data file 9 Provided are details of previously reported transcriptome datasets used in data comparison. Click here for file

Additional data file 10 Described is the methodology employed for transcriptional profiling by oligonucleotide array. Click here for file

Additional data file 11 Listed are the primers used for mutant genotyping, probes for mRNA *in situ *hybridization and RT-PCR. Click here for file
