# Supplementary material for: Genetic subtraction profiling identifies genes essential for Arabidopsis reproduction and reveals interaction between the female gametophyte and the maternal sporophyte
Source: Genome Biol. 2007 Oct 3;8(10):R204. doi: 10.1186/gb-2007-8-10-r204 (PMC2246279; doi:10.1186/gb-2007-8-10-r204)
Supplement: Additional data file 1 — Presented is a table listing the gene validation for embryo sac expression. [file gb-2007-8-10-r204-S1.pdf]

Additional data file 1. Validation for embryo sac expression

TAIR ID	Gene Description	FC[a]	Embryo Sac Expression (Method)[b]	Reference
(i) *coatlique* Dataset				
At4g37450	AGP18	1.50	egg, synergids, antipodals (ET, ISH)	[87]
At1g80370	Cyclin A2;4, Endocycle Regulation	1.28	embryo sac (PRG)	This study
At4g01970	Galactosyltransferase	1.51	embryo sac (ET)	This study
At5g40260	Nodulin MtN3 Protein	1.99	embryo sac (ISH, PRG)	This study; [34]
At4g30590	Plastocyanin	1.83	embryo sac (ISH, PRG)	This study; [34]
At4g34110	Polyadenylate-Binding Protein 2 (PAB2)	1.30	embryo sac (PRG)	[88]
At1g71770	Polyadenylate-Binding Protein 5 (PAB5)	1.38	embryo sac (PRG)	[89]
At1g78940	Protein Kinase, Cell Cycle Progression	1.35	embryo sac (ISH)	This study
At1g28220	Purine Permease 3 (PUP3)	1.30	synergids (PRG)	This study
At5g60270	Receptor Kinase	1.56	egg cell (ISH)	This study
At1g58470	RNA Binding Protein, RPB1	1.34	embryo sac (PRG)	[90]
At1g24260	SEPALLATA 3 (SEP3)	1.34	embryo sac (ISH)	[91]
At3g61160	Shaggy-Like Kinase β (ASKβ)	1.32	central cell (ISH)	[27]
At5g50915	TCP Transcription Factor	1.36	egg, central cell (ISH)	This study
At3g61740	Trithorax Like Protein (ATX3)	1.47	central cell, egg, synergids (ISH)	This study
(ii) sporocyteless Dataset				
At1g02580	E(Z) Homologue, MEDEA	1.64	egg, central cell (PRG, ISH)	[85]
At2g35670	Fertilization Independent Seed 2 (FIS2)	4.31	central cell (PRG)	[25]
At4g02060	MADS Box Protein, PROLIFERA	1.34	embryo sac (ET, ISH)	[15]
At4g18770	R2R3-MYB Transcription Factor (MYB98)	14.23	synergids (PRG, PrGFP)	[22]
At4g25530	Homeodomain Protein, FWA	5.04	central cell (PrGFP)	[92]
At2g37560	Origin recognition complex subunit 2 (ORC2)	1.28	egg, synergid, central cell (ISH)	[93]
At5g40260	Nodulin MtN3 Protein	14.22	embryo sac (ISH, PRG)	This study; [34]
At4g30590	Plastocyanin	1.83	embryo sac (ISH, PRG)	This study; [34]
At1g71770	Polyadenylate-binding protein 5 (PAB5)	1.38	embryo sac (PRG)	[89]
At1g26795	Self-Incompatibility Protein-Related	25.98	embryo sac (PRG)	[34]
At1g36340	E2, Ubiquitin-Conjugating Enzyme	14.97	antipodals (PRG)	[34]
At3g24220	9-Cis-Epoxycarotenoid Dioxygenase (NCED6)	2.24	central cell (PrGFP)	[94]

[a]Genes that exhibited a greater than 1.28-fold change (FC) in their wild-type signal values from the corresponding values from mutants that lacked an embryo sac ($P < 0.1$)

[b]Gene expression was identified by enhancer-traps (ET), gene promoter elements fused with marker genes such as β-glucuronidase (PRG) or GFP (PrGFP), and *in situ* hybridization (ISH)
