# Supplementary material for: Genetic subtraction profiling identifies genes essential for Arabidopsis reproduction and reveals interaction between the female gametophyte and the maternal sporophyte
Source: Genome Biol. 2007 Oct 3;8(10):R204. doi: 10.1186/gb-2007-8-10-r204 (PMC2246279; doi:10.1186/gb-2007-8-10-r204)
Supplement: Additional data file 4 — Presented is a list of genes from this work that were previously identified as being essential for reproductive development. [file gb-2007-8-10-r204-S4.pdf]

**Additional data file 4.** Genes essential for female gametogenesis, fertilization and seed development are present in the embryo sac transcriptome datasets

| Locus Name | Functional Description | FC[a] | Developmental Function | Reference |
|---|---|---|---|---|
| (i) *coatlique* Dataset | | | | |
| *KERRIDWIN* | Auxin-Responsive GH3 Family Protein | 1.29 | fusion of polar nuclei | This study |
| *CMT3* | Chromomethylase 3 | 1.29 | embryo & endosperm | [63] |
| *AGP18* | Arabinogalactan-Protein 18 | 1.50 | female gametogenesis | [87] |
| *HOG1* | S-Adenosyl Homocysteine Hydrolase | 1.29 | embryo & endosperm | this study; [55] |
| *FREYA* | RNA Helicase | 1.36 | embryo | SeedGenes[b]; this study |
| *OMISHA* | N-Terminal Acetyltransferase | 1.30 | embryo | SeedGenes; this study |
| *EMB1381* | Unknown | 1.28 | embryo | SeedGenes |
| *ILITHYIA* | Translational Activator Family Protein | 1.30 | embryo | This study |
| *FUS2* | Repressor of Photomorphogenesis | 1.31 | seed | [96] |
| *FUS6* | COP9 Signalosome Complex Subunit 1 | 1.35 | seed | [97] |
| *(ii) sporocyteless Dataset* | | | | |
| *MEA* | *Arabidopsis* Homolog of E(Z) | 1.64 | embryo & endosperm[c] | [12] |
| *FIS2* | Fertilization-Independent Seed 2 Protein | 4.31 | embryo & endosperm[c] | [11] |
| *PRL* | DNA Replication Licensing Factor | 1.34 | embryo & endosperm | [15] |
| *MYB98* | R2R3-MYB Transcription Factor | 3.73 | synergid differentiation | [22] |
| *TT16* | MADS-domain Protein | 1.71 | seed | [98] |
| *TTG2* | WRKY Family Transcription Factor | 1.79 | seed | [99] |
| *FWA* | Homeodomain Protein | 2.41 | endosperm | [92] |
| *CUC2* | Cup Shaped Cotyledon 2 | 2.66 | embryo | [100] |
| *EMB2220* | CCAAT-Binding Transcription Factor | 1.34 | female gametogenesis & embryo | SeedGenes |
| *EDA40* | C3HC4-type RING finger family protein | 1.53 | fusion of polar nuclei | [13] |
| *CMT3* | Chromomethylase 3 | 1.40 | embryo & endosperm | [63] |
| *ORC2* | Origin Recognition Complex Subunit 2 | 1.28 | embryo & endosperm | [93] |
| *EMB2219* | Transcription Termination Factor | 1.58 | embryo | SeedGenes |
| *δVPE* | Asparaginyl Endopeptidase | 1.49 | integument & embryo | [101] |
| *YDA* | MAP3K Protein Kinase | 1.59 | embryo | [102] |
| *PDE317* | DEAD/DEAH Box Helicase | 1.28 | embryo | SeedGenes |
| *EMB3005* | Cysteine Proteinase RD19a | 1.30 | embryo | SeedGenes |
| *EMB1011* | Proline-Rich Family Protein | 1.28 | embryo | SeedGenes |
| *EMB175* | PPR Repeat-Containing Protein | 1.41 | embryo | [103] |
| *EMB1270* | PPR Repeat-Containing Protein | 1.28 | embryo | SeedGenes |
| *EMB2221* | WD-40 Repeat Family Protein | 1.40 | embryo | SeedGenes |
| *UNE9* | Cytochrome P450 | 1.89 | pollen tube attraction | [13] |
| *EDA23* | LRR Protein Kinase | 2.53 | female gametogenesis | [13] |
| *MEE19* | En/Spm Transposon Protein | 9.92 | endosperm | [13] |
| *MEE23* | FAD-Binding Protein | 2.29 | endosperm | [13] |
| *EMB1353* | Unknown | 2.12 | embryo & endosperm | SeedGenes |

[a]Genes that exhibited a greater than 1.28-fold change (FC) in their wild-type signal values from the corresponding values from mutants that lacked an embryo sac ($P < 0.1$)

[b]An *Arabidopsis* NSF 2010 project on essential genes for seed development [95]

[c]Maternal effect – *FERTILIZATION INDEPENDENT SEED* (*FIS*) class of genes
