# Supplementary material for: Genetic subtraction profiling identifies genes essential for Arabidopsis reproduction and reveals interaction between the female gametophyte and the maternal sporophyte
Source: Genome Biol. 2007 Oct 3;8(10):R204. doi: 10.1186/gb-2007-8-10-r204 (PMC2246279; doi:10.1186/gb-2007-8-10-r204)

Additional data file 6. An overview of gene discovery by three independent methods across two types of datasets from two mutants. (A) A histogram depicting the total number of genes found in four datasets using three data analysis packages such as GeneSpring, dCHIP and gcRMA. (B) A Venn diagram showing the number of genes that are unique to, and overlapping across, three statistical analyses within the embryo sac gene list. (C) A Venn diagram showing the number of genes that are unique to, and overlapping across, three statistical analyses within the sporophytic gain of expression datasets. In B and C, the numbers N;N represent the corresponding number of genes in *coa* and *spl* datasets

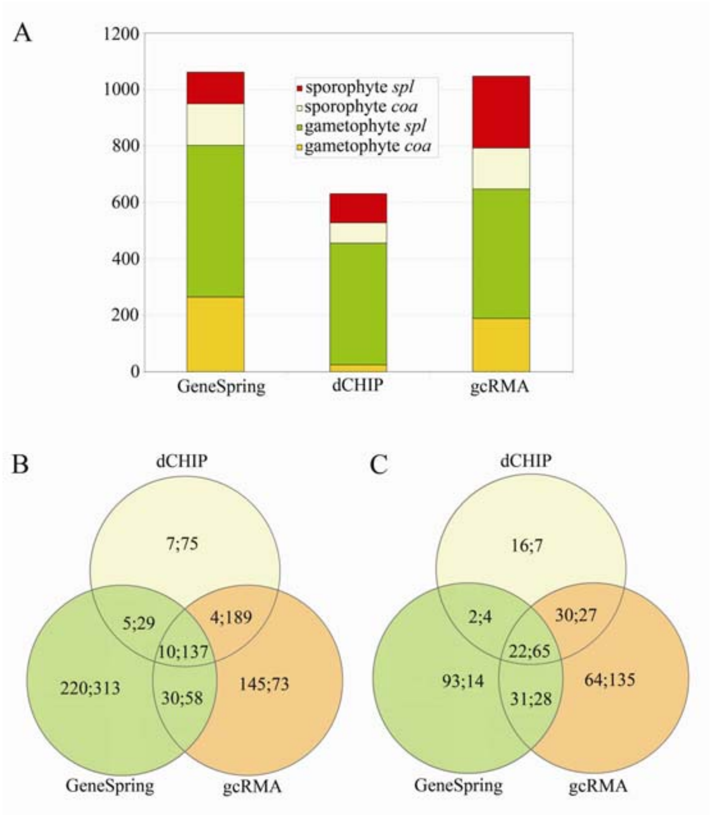

Supplement: Additional data file 6 — Illustrated is the scale of gene discovery by three independent methods across two types of datasets from two mutants. [file gb-2007-8-10-r204-S6.pdf]
