# Supplementary material for: Genetic subtraction profiling identifies genes essential for Arabidopsis reproduction and reveals interaction between the female gametophyte and the maternal sporophyte
Source: Genome Biol. 2007 Oct 3;8(10):R204. doi: 10.1186/gb-2007-8-10-r204 (PMC2246279; doi:10.1186/gb-2007-8-10-r204)
Supplement: Additional data file 7 — Presented is a table summarizing gene identities and the statistical treatments, confirming the necessity of different statistical treatments to identify embryo sac expressed genes. [file gb-2007-8-10-r204-S7.pdf]

Additional data file 7. Different statistical treatments are necessary to identify embryo sac – expressed and/or essential genes

Probe ID	Gene ID	Analysis	FC
***coatlique* dataset**			
251276_at	At3g61740	G	1.47
253050_at	At4g37450	G	1.50
251358_at	At3g61160	G	1.32
264139_at	At1g78940	G	1.35
253280_at	At4g34110	G	1.30
261511_at	At1g71770	G	1.38
245808_at	At1g58470	G	1.31
262859_at	At1g64790	G	1.30
251311_at	At3g61140	G	1.35
255550_at	At4g01970	G	1.51
245356_at	At4g13940	G	1.29
245661_at	At1g28220	G	1.30
266465_at	At2g47750	G	1.29
260282_at	At1g80410	G	1.29
264087_at	At2g31340	G	1.28
260417_at	At1g69770	G	1.29
249401_at	At5g40260	G, dC, R	1.99
253634_at	At4g30590	G, dC, R	1.83
264872_at	At1g24260	G, R	1.34
248460_at	At5g50915	G, R	1.36
247617_at	At5g60270	G, R	1.56
260329_at	At1g80370	G, R	1.28
250273_at	At5g13010	R	1.36
255799_at	At4g10180	R	1.31

Probe ID	Gene ID	Analysis	FC
***sporocyteless* dataset**			
265801_at	At2g35670	dC	4.31
260908_at	At1g02580	dC	1.64
267414_at	At2g34790	dC	2.29
245239_at	At4g25530	dC, R	2.41
254619_at	At4g18770	dC, R	3.73
249013_at	At5g44700	dC, R	2.53
260124_at	At1g36340	dC, R	14.87
257242_at	At3g24220	dC, R	2.24
249401_at	At5g40260	dC, R	14.42
253634_at	At4g30590	dC, R	12.21
256719_at	At2g34130	dC, R	9.92
261271_at	At1g26795	dC, R	25.98
253656_at	At4g30090	dC, R	2.12
250320_at	At5g12840	G	1.34
260417_at	At1g69770	G	1.40
264748_at	At1g70070	G	1.28
255513_at	At4g02060	G	1.34
260291_at	At1g63700	G	1.59
267173_at	At2g37560	G	1.28
250893_at	At5g03800	G	1.41
263899_at	At2g21710	G	1.58
258149_at	At3g18110	G	1.28
261511_at	At1g71770	G	1.33
253011_at	At4g37890	G	1.53
252927_at	At4g39090	G	1.30
265074_at	At1g55540	G	1.28
251179_at	At3g63460	G	1.40
258918_at	At3g10560	G, dC	1.89
249851_at	At5g23260	G, dC, R	1.69
248240_at	At5g53950	G, dC, R	2.74
257130_at	At3g20210	G, dC, R	1.49
265954_at	At2g37260	G, R	1.79

G=GeneSpring; dC=dCHIP; R=gcRMA. FC=Fold Change

Note: Refer to Additional data files 1 and 4 for description of genes
