# Supplementary material for: Genetic subtraction profiling identifies genes essential for Arabidopsis reproduction and reveals interaction between the female gametophyte and the maternal sporophyte
Source: Genome Biol. 2007 Oct 3;8(10):R204. doi: 10.1186/gb-2007-8-10-r204 (PMC2246279; doi:10.1186/gb-2007-8-10-r204)
Supplement: Additional data file 8 — Listed are the identifiers of maize and wheat ESTs from the embryo sac cell types, which were used in BLAST analysis of Arabidopsis proteins. [file gb-2007-8-10-r204-S8.pdf]

Additional data file 8. List of maize and wheat ESTs from the embryo sac cell types, which were used in BLAST analysis of *Arabidopsis* proteins

EST-<br>Tissue Source	Number of ESTs	Number of Represented Genes[c]	GenBank ID[a]	Reference
Embryo sac	10,747	6,032 (4,869)	Several NCBI IDs (M)	[59]
Egg cell	5,925	3,761 (3,233)	Several NCBI IDs (M)<br>DN591121 - DN591151 (M)<br>AL830671 - AL831324 (W)<br>CV973579 - CV973658 (W)	[59]<br>[54]<br><br>[58]
Central cell and endosperm 6DAP	15,677	5,853 (4,321)	DN591090-DN591120 (M)<br>Several NCBI IDs[b] (M)	[59]<br>[104]

[a]M = maize; W = wheat
[b]EST data were included based on the 6DAP library names (Endosperm_2, Endosperm_4, Endosperm_5, Endosperm_6: refer to [104] for details)
[c]The sequences of each EST data set were clustered using blastclust (NCBI) to get estimates of the number of independent clusters (=represented genes). Number of singletons is given in brackets
