# Supplementary material for: Genetic subtraction profiling identifies genes essential for Arabidopsis reproduction and reveals interaction between the female gametophyte and the maternal sporophyte
Source: Genome Biol. 2007 Oct 3;8(10):R204. doi: 10.1186/gb-2007-8-10-r204 (PMC2246279; doi:10.1186/gb-2007-8-10-r204)
Supplement: Additional data file 9 — Provided are details of previously reported transcriptome datasets used in data comparison. [file gb-2007-8-10-r204-S9.pdf]

Additional data file 9. Details of previously published transcriptome datasets used in data comparison

Transcriptome ID	Tissue type	Replicated?	Reference
ATGE 01 A,B,C	cotyledon	triplicate	[28][a]
ATGE 02 A,B,C	hypocotyl	triplicate	[28][a]
ATGE 03 A,B,C	root	triplicate	[28][a]
ATGE 04 A,B,C	leaf shoot apex	triplicate	[28][a]
ATGE 05 A,B,C	leaf	triplicate	[28][a]
ATGE 06 A,B,C	shoot apex	triplicate	[28][a]
ATGE 07 A,B,C	shoot	triplicate	[28][a]
ATGE 08 A,B,C	inflorescence shoot	triplicate	[28][a]
ATGE 09 A,B,C	root	triplicate	[28][a]
ATGE 10 A,B,C	young leaf	triplicate	[28][a]
ATGE 12 A,B,C	mature leaf	triplicate	[28][a]
ATGE 13 A,B,C	mature leaf	triplicate	[28][a]
ATGE 14 A,B,C	mature leaf	triplicate	[28][a]
ATGE 15 A,B,C	young leaf	triplicate	[28][a]
ATGE 16 A,B,C	young leaf	triplicate	[28][a]
ATGE 17 A,B,C	young leaf	triplicate	[28][a]
ATGE 19 A,B,C	petiole	triplicate	[28][a]
ATGE 25 A,B,C	senescent leaf	triplicate	[28][a]
ATGE 26 A,B,C	cauline leaf	triplicate	[28][a]
ATGE 27 A,B,C	internode shoot	triplicate	[28][a]
ATGE 28 A,B,C	nodeshoot	triplicate	[28][a]
ATGE 34 A,B,C	sepal	triplicate	[28][a]
ATGE 35 A,B,C	petal	triplicate	[28][a]
ATGE 40 A,B,C	pedical	triplicate	[28][a]
ATGE 41 A,B,C	sepal	triplicate	[28][a]
ATGE 42 A,B,C	petal	triplicate	[28][a]
ATGE 79 A,B,C	seed (torpedo embryo)	triplicate	[28][a]
ATGE 81 A,B,C	seed (walking stick)	triplicate	[28][a]
ATGE 82 A,B,C	seed (early curled cot	triplicate	[28][a]
ATGE 83 A,B,C	seed (green cotyledon)	triplicate	[28][a]
ATGE 84 A,B,C	seed (green cotyledon)	triplicate	[28][a]
ATGE 87 A,B,C	early rosette	triplicate	[28][a]
ATGE 89 A,B,C	mid rosette	triplicate	[28][a]
ATGE 90 A,B,C	late rosette	triplicate	[28][a]
ATGE 73 A,B,C	pollen	triplicate	[28][a]
ATGE 76 A,B,C	silique (globular embryo)	triplicate	[28][a]
ATGE 77 A,B,C	silique (heart embryo)	triplicate	[28][a]
ATGE 78 A,B,C	silique (triangle embryo)	triplicate	[28][a]
unicellular	pollen	duplicate	[31,32][b]
bicellular	pollen	duplicate	[31,32][b]
tricellular	pollen	duplicate	[31,32][b]
mature pollen	pollen	triplicate	[33][c]

[a] downloaded from TAIR [82]
[b] downloaded from NASC [105]
[c] downloaded from Array Express [106]
