# Supplementary material for: Genetic subtraction profiling identifies genes essential for Arabidopsis reproduction and reveals interaction between the female gametophyte and the maternal sporophyte
Source: Genome Biol. 2007 Oct 3;8(10):R204. doi: 10.1186/gb-2007-8-10-r204 (PMC2246279; doi:10.1186/gb-2007-8-10-r204)
Supplement: Additional data file 10 — Described is the methodology employed for transcriptional profiling by oligonucleotide array. [file gb-2007-8-10-r204-S10.pdf]

Additional data file 10. Transcriptional profiling by oligonucleotide array: methods

Material Harvest

We isolated the pistils at a stage between late 11 to late 12 floral stages [39]. The gynoecia were dissected out of the flowers with a syringe needle (BD Micro-Fine, Franklin Lakes, USA) under a dissection scope (Leica Microsystem, Bensheim, Germany) and placed into a RNA protecting solution (RNA-Later, Ambion Inc., Austin, USA). In order to get statistically relevant data, three replicate tissue samples of wild-type and *coa* were harvested and extracted. Each tissue sample represented approximately 100 to 200 gynoecia, which yielded on average of 30 µg total RNA, a sufficient amount for the Affmetrix Gene Chip© hybridization procedure (manufacturer's instructions).

Transcriptome Analysis

RNA was extracted from the gynoecia using TRIZOL according to the manufacturer's instructions (Invitrogen-Life Technologies, Paisley, UK). The RNA was purified on an RNA column (RNAeasy, Qiagen, Basel Switzerland) according to the manufacturer's instructions. At least 15 µg of each total RNA sample (each sample is hybridized on a separate chip) was used to start the cDNA synthesis. Total RNA was processed into cDNA using the Superscript Double-Stranded cDNA Synthesis Kit© (Invitrogen) and a special primer 5-GGC CAG TGA ATT GTA ATA CGA CTC ACT ATA GGG AGG CGG (dT)24-3. The purified cDNA (GenChip Columns, Affymetrix, Santa Clara, CA) was used in an *in vitro* transcription reaction in the presence of 2 mM biotin-11-CTP and biotin-16-UTP (ENZO Life Sciences, Farmingdale, NY) with the MegaScript High Yield Transcription Kit© (Ambion, Austin, TX). Approximately 15 µg of each cRNA was fragmented with a fragmentation-buffer (GeneChip Cleanup, Affymetrix), mixed with the hybridization cocktail and hybridized on to an ATH1 gene chip according to the standard protocols from Affymetrix Inc. All hybridizations, three replicates each of wild type and *coa*, were hybridized and processed at the same time as recommended by the manufacturer (Affymetrix).

Bio-Informatic Analysis

The raw microarray data from two mutants and the corresponding wild-type in triplicate were analysed using three independent packages: i) GeneSpring; ii) dCHIP; and iii) gcRMA using bayesian approach. The raw data were normalized using Affymetrix Microarry Suite© 5.0, and an algorithm was set to determine absolute calls for each probe (flag P: present; M: marginal; and A: absent). The data were analyzed with the GeneSpring version 7.0 (Silicon Genetics, San Carlos, CA). Using a per-chip 50th percentile method, each chip was normalized on the median, allowing comparison among chips. Subsequently, a per-gene normalization was performed, which normalizes the expression of every gene on its mean among the three replicates. Internal controls were performed on the raw data for the housekeeping genes Glyceraldehyde-3-phosphate dehydrogenase C (GAPc, At3g04120), Poly-ubiquitin *10* (UBQ10, At4g05320) and ACTIN *7* (ACT7, At5g09810).

Genes with low control value C were omitted from further downstream analysis. Genes with an "absent" call for the detection value in all replicates of one gene chip experiment were excluded from the list (flag filtering). Statistical analyses were performed on normalized values and examined as a log ratio to obtain a normally distributed population for parametric analysis. Differentially expressed genes were identified by computing student's t-test based on equal variances. P value was set to < 0.1, and probes that exhibited a larger than 1.28-fold differences in their wild-type signal values in comparison to the mutant values were

designated embryo sac–expressed. When the probes from the mutant datasets showed a larger than two-fold difference than their wild-type values, they were extracted as sporophytic datasets.

For the second method, we used the DNA-Chip Analyzer (dCHIP) Version Release (Sep 23, 2005) [107]. The arrays were normalized per dataset to a baseline array with median CEL intensity of 150 by applying an Invariant Set Normalization Method [45]. Normalized CEL intensities of all the arrays were used to obtain model-based gene expression indices based on a PM (Perfect Match)-only model [108]. Replicate data for the same sample type were weighted gene-wise by using inverse squared standard error as weights. Only genes called Present at least once in two of the three replicates per condition, and genes within replicate arrays called Present within a variation of $0 <$ Median (Standard Deviation/Mean) < 0.5, were kept for downstream analysis. Thus, genes with highly inconsistent expression levels within replicate arrays were excluded. All genes compared were considered to be differentially expressed if the 90% lower confidence bound of the fold change between experiment and baseline was above the set cut-off (as discussed above).

For the analysis using an empirical bayesian approach, background correction, normalization and feature extraction were performed using the gcRMA algorithm [109] implemented in the Bioconductor software version 2.3.0 [79]. The algorithm uses a model-based background correction that takes probe sequence and GC-content into account, a quantile normalization procedure and a robust linear model for signal calculation. Preprocessed signals were statistically analyzed using an empirical bayesian approach implemented in the Bioconductor package limma [110]. Resulting p-values were corrected for multiple testing using the q-value package [111] and a false discovery rate (fdr) was calculated for each gene. Genes with a fdr $<$ 0.05 were considered to be differentially expressed. Subsequently, we calculated the fold-change as discussed above for the first method.
