# Supplementary material for: Genetic subtraction profiling identifies genes essential for Arabidopsis reproduction and reveals interaction between the female gametophyte and the maternal sporophyte
Source: Genome Biol. 2007 Oct 3;8(10):R204. doi: 10.1186/gb-2007-8-10-r204 (PMC2246279; doi:10.1186/gb-2007-8-10-r204)
Supplement: Additional data file 11 — Listed are the primers used for mutant genotyping, probes for mRNA in situ hybridization and RT-PCR. [file gb-2007-8-10-r204-S11.pdf]

Additional data file 11. Primers used for mutant genotyping, probes for mRNA *in situ* hybridization and RT-PCR

Gene/Mutant	Purpose[1]	Primers	T_m[2]	Comments
At1G78940	ISH (ES)	GCA GGA AAT CAA GGA AAG TC + CCG TTA TGG GCC TAA CA	52	
At5g40260	ISH (ES)	TGG TGT TGG GTT AGT TAT CGA + TTC TCC TAA ACC CTC TCC GTA	58	
At4g30590	ISH (ES)	CCT TGT CTT CCT CTT GTT TGC + CCA AAC CAA CCA CGA CTG C	54	
At3G61740	ISH (ES)	CTG CAG CAA GAT GCC GTA + GCC GTT CTC TGA ATG ATG	52	
At5g50915	ISH (ES)	CTA CAC CTC ACT CCT CCA + GTG TTG GTG ATG GCT GAT GGT C	55	
At5G60270	ISH (ES)	CAG CAC CAC TCG TGA TCC + CGT TCT ACA AGC TAA GCT	52	
At3G12110	ISH (SP)	AAC TTT CAA CAC TCC TGC CAT G + CTG CAA GGT CCA AAC GCA GA	60	
At4G12410	RT, ISH (SP)	AAC ACA GAG AGA GAT AC + GCT AAT GAC CGG ATT CC	50	
At1G75580	RT, ISH (SP)	GTG TAC CCT GTG AGA GAC TTT + AAA GTC TCT CAC AGG GTA CAC	56	
At5G03200	RT, ISH (SP)	GAG GCA ACT CCA GCG GAA G + TGC GCA CCC GCT ACA CAT AC	60	
At5G15980	RT, ISH (SP)	CCA ACG AAT CTG CTG TAG + CAG ATG ATA AGC AAG TGG	50	
STM	RT, ISH (SP)	CAA CGT GTC GAG TGT CAA TTC + GTC CAG CCC CGT TGA TTC	55	
At5G45420	RT	CAC GAT GAG TCA TCC ACG + GCC ACC GTC TCC CAT CTC	52	
At3G16770	RT	GAT GAT GTC ATT GCG TCG + CTA CAC ATT ACA CAA CAG AC	52	
At3G55660	RT	ATG GAG GAT AAT AGC TGT ATC GGG + CCA ATT ATC TCC GGG GTT GA	55	
At4G15800	RT	ACT CTC CAC AAA ACC CGT TG + GTC GTG AGC AAG GAA CTG TG	55	
At1G25330	RT	GTA GTG TCT CTA ATG GCA CG + TCT CTT CGA CCC ACT CTC	50	
SUP	RT	CTC TAA GAG ACA GAC AGA CAT AG + GGC CAT GAA AAC CCT AGA AGA T	52	
kerridwin-1	genotyping	SM32: TAC GAA TAA GAG CGT CCA TTT TAG AGT GA	55	SM32 + B8-R = 400 bp
		B8-F: GGC TGA AAC ATC AAC TCC TTG TG		
		B8-R: GCA CAT GTT AGG CAA GAG AGT G	57	B8-f + B8-R = 500 bp
frigg-1	genotyping	SK.LB: AGC TGT TGC CCG TCT CAC	55	S1F1 + S1R1 = 263 bp
		S1F1: CCT GGC ATG TTT GCT CTT CG		
		S1R1: TGC AAG TGT GCT TCC GAG ATG	58	S1F1 + SK.LB = 300 bp
freya-1	genotyping	VER-RB1: CAT CGG GAA TCG AAA GAT C	47	VER-RB1 + FEY-R: 500 bp
		FEY-F: CAA GTC ATG TTG CTG TCG ATA A		
		FEY-R: CGG AAC AAA GGA TCA CCG C	52	FEY-F + FEY-R = 268 bp
omisha-1	genotyping	SYN-LB1: CAG TAC ATT AAA AAC GTC CGC AA	52	SYN-LB1 + OMA-F = 400 bp
		OMA-F: GGT ATC TCT CTT GTC AAA GGT TG		
		OMA-R: GCA CTG ATA TGC CTT ATC TCA	52	FEY-F + FEY-R = 445 bp
ilithyia-1	genotyping	SK.LB: AGC TGT TGC CCG TCT CAC	55	SK.LB + ILA-R = 280 bp
		ILA-F: GTG CAG GCT GAT CCC AAC		
		ILA-R: CTC TTC ATC AGA TGC CTA CAT G	52	ILA-F + ILA-R = 242 bp

[1]ISH = mRNA *in situ* hybridisation; ES = embryo sac dataset; SP = sporophyte dataset; RT = Reverse-transcriptase PCR (RT-PCR)
[2]T_m = melting temperature ($\circ$C)
